# Hydrogels with intrinsic antibacterial activity prepared from naphthyl anthranilamide (NaA) capped peptide mimics

**DOI:** 10.1038/s41598-022-26426-1

**Published:** 2022-12-23

**Authors:** Vina R. Aldilla, Renxun Chen, Rajesh Kuppusamy, Sudip Chakraborty, Mark D. P. Willcox, David StC. Black, Pall Thordarson, Adam D. Martin, Naresh Kumar

**Affiliations:** 1 School of Chemistry, The University of New South Wales, Sydney, NSW 2052 Australia; 2 School of Optometry and Vision Science, The University of New South Wales, Sydney, NSW 2052 Australia; 3 Faculty of Medicine and Health Sciences, Dementia Research Centre, Macquarie University, Sydney, NSW 2109 Australia

**Keywords:** Gels and hydrogels, Self-assembly, Antimicrobials

## Abstract

In this study, we prepared antibacterial hydrogels through the self-assembly of naphthyl anthranilamide (NaA) capped amino acid based cationic peptide mimics. These ultra-short cationic peptide mimics were rationally designed with NaA as a capping group, l-phenylalanine, a short aliphatic linker, and a cationic group. The synthesized peptide mimics efficiently formed hydrogels with minimum gel concentrations between 0.1 and 0.3%w/v. The resulting hydrogels exhibited desirable viscoelastic properties which can be tuned by varying the cationic group, electronegative substituent, or counter anion. Importantly, nanofibers from the NaA-capped cationic hydrogels were found to be the source of hydrogels’ potent bacteriacidal actvity against both Gram-positive and Gram-negative bacteria while remaining non-cytotoxic. These intrinsically antibacterial hydrogels are ideal candidates for further development in applications where bacterial contamination is problematic.

## Introduction

The self-assembly of low molecular weight gelators (LMWGs), into supramolecular hydrogels is an attractive method to generate biocompatible materials. LMWGs based on short peptides or peptidomimetics, that consist of less than six amino acid (AA) sequences, have gained a lot of attention due to their tunable supramolecular properties, potential biocompatibility, and cost-effective synthesis when compared to natural peptides^1–4^. This class of LMWGs were reported to form supramolecular hydrogels via non-covalent interactions such as combination of H-bonding, π–π stacking, and hydrophobic interactions^5^. The N-terminus of peptides based LMWGs are often modified by incorporating a non-proteinaceous aromatic groups as a capping group, which can enhance the π–π stacking capability and drive the self-assembly process leading to hydrogel formation^1^. Apart from the most widely used capping group, fluorenylmethoxycarbonyl (Fmoc), various other aromatic group including naphthalene^6,7^, indole^8^, benzimidazole^9^, pentafluorobenzene^10^, pyrene^11^, and others^12^ have been reported to induce hydrogelation.

Antibacterial hydrogels based on short-peptides have been reported^13^. The majority of examples require encapsulation of another active agent (e.g. antibiotics, silver ion, or salicylic acid) in order to achieve their antibacterial efficacy^14–21^. The need for encapsulation of active agents can lead to problems associated with antimicrobial resistance, loading capacity, release rate, and biodegradability. Hence, hydrogels with inherent antibacterial properties are highly desirable for clinical use.

Limited examples of short peptide-based hydrogels with inherent antibacterial properties have been reported in literature. For instance, Fmoc-diphenylalanine and Fmoc-capped short peptides bearing either lysine-rich or pyridinium groups have been found with moderate antibacterial activities against Gram-positive and Gram-negative bacteria^22,23^. In addition, naphthalene-capped tetrapeptides displayed significant activity against *S. epidermis* and *E. coli* biofilm^24^.

Recently, we have demonstrated that anthranilamide-based diphenylalanine peptide mimics formed self-assembled hydrogels with promising antibacterial activity against *S. aureus*^25^. Unlike Fmoc-based LMWGs, the anthranilamide-based peptide mimics have diverse chemical modifications that can be introduced to the scaffold, such as hydrophobic groups, substitutions on aromatic caps, and varying chain lengths, cationic/anionic charges etc. This base scaffold provides a versatile platform for the systematic examination of the effects of different chemical moieties on intramolecular interactions, that induce self-assembly and gel formation, and on biological functions such as antimicrobial activity.

**Figure 1:**
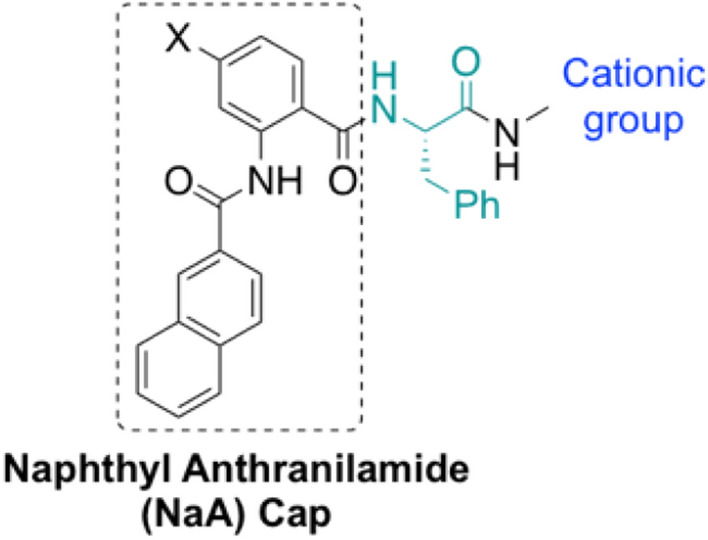
General structure of naphthyl anthranilamide (NaA)-capped ultra-short hydrogelators.

In this work, we present the hydrogel formation of a library of naphthyl anthranilamide (NaA) capped amino acid-based cationic peptide mimics. These ultra-short cationic peptide mimics composed of a NaA capping group, l-phenylalanine, a short aliphatic linker, and a cationic group (Fig. 1). These peptide mimics could be facilely synthesized on a gram scale via ring-opening reaction of isatoic anhydride in solution phase^25,26^, rather than using solid phase peptide synthesis (SPPS). As an effort to reduce the toxicity of the previously reported anthranilamide-based hydrogels, the design of current hydrogelators involves only one unit of phenylalanine. Apart from providing *π*–*π* stacking interaction, the aromatic side chain of phenylalanine also increases the overall hydrophobicity of the gelators.

Cationic charge was also introduced by incorporating either ammonium or guanidinium groups, which are attached through a short aliphatic linker, on the C-terminus of phenylalanine. The presence of cationic charge was also envisaged to aid the solubility of the resulting hydrogelators. Moreover, the cationic groups employed, have been reported to be responsible for antibacterial activity of AMPs and their mimics^27–29^. The antibacterial efficacy of the cationic hydrogels was evaluated in vitro against *S. aureus* and *E. coli*.

The structural, morphological, and mechanical properties of the cationic hydrogels were investigated using various techniques, namely ^1^H NMR, circular dichroism (CD) spectroscopy, attenuated total reflectance infrared (ATR-FTIR), atomic force microscopy (AFM), and rheometer. The cytotoxicity was examined on HEK293T cells.

## Result and discussion

### NaA-capped ultra-short cationic hydrogelators design rationale

**Figure 2:**
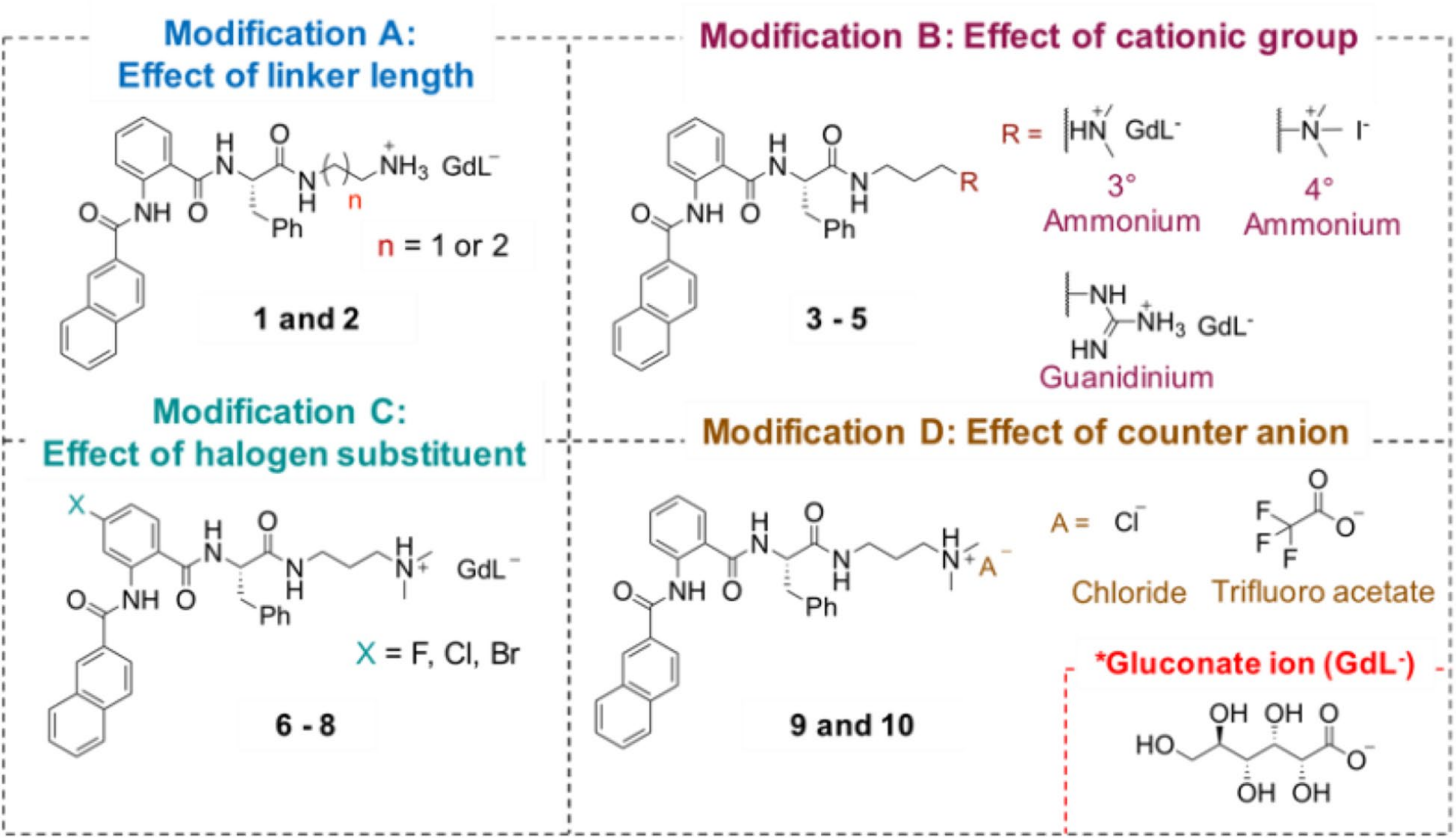
Hydrogelators **1**–**10** derived from NaA-capped cationic peptide mimics.

The NaA-capped cationic hydrogelators reported herein were systematically designed and synthesized with four modifications (Fig. 2), such as varying the linker length (Modification A), altering the cationic moiety (Modification B), introducing halogen (i.e. fluoro, chloro, and bromo) substituent (Modification C), and changing the counter anion (Modification D). The effect of each modification to the hydrogelation capability, mechanical properties, and antibacterial activity of the resulting hydrogels were evaluated.

As describe in Scheme S1, these peptide mimics were synthesized in excellent yields (60–78%).The NaA-capped cationic peptide mimics were synthesized on a gram scale via ring-opening reaction of isatoic anhydride derivatives using l-phenylalanine followed by amide coupling reaction to introduce the naphthyl moiety^25,26^. The cationic charge within these peptide mimics were introduced by either adding 1.5 equivalent of either glucono-∂-lactone (GdL), during the hydrogel formation, or treating the intermediate **3a** with dilute acids (TFA or HCl).

### Hydrogelation studies

**Figure 3:**
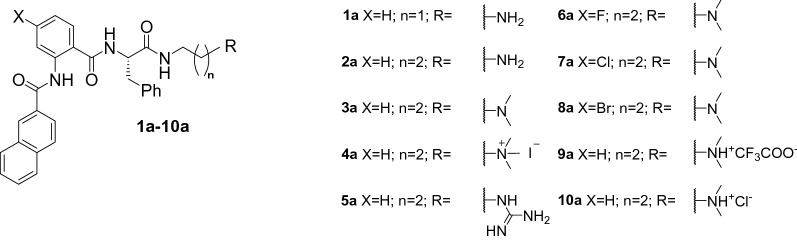
Structure of peptide mimic hydrogelators used for hydrogelation studies.

The majority of the cationic peptide mimics formed hydrogels at physiological pH using a combination of physical and chemical triggers which could alter their thermodynamic equilibrium. Addition of GdL (1.5 equivalent) to compound **1a–8a** (Fig. 3) and mild heating are required to generate the ammonium group, present in hydrogels **1**–**8**. The presence of cationic group would lead to dissolution of these compounds in water (Scheme S2). Meanwhile, the cationic peptide mimics **9a** and **10a** (Fig. 3), bearing TFA^−^ and Cl^−^ as counter anion, were readily soluble in water after mild heating was applied to form hydrogels **9** and **10**. All solutions turned clear upon heating, indicating complete disolution. Upon cooling to room temperature, the solutions became viscous within 3 h (Fig. S1).

To form self-supporting hydrogels, the addition of NaCl (5.0 equivalent) was attempted. The majority of viscous solutions of the cationic peptide mimics became solid-like hydrogels after addition of NaCl as observed through vial inversion test. Self-supporting characteristic of hydrogels were observed from peptide mimics **2a**, **3a**, **4a**, **6a**, **7a**, **9a**, and **10a**. In contrast, syneresis, a phenomenon where water was expelled followed by structural disintegration^30–32^, was observed for hydrogels **1**, **5** and **8**, made from corresponding peptide mimics **1a** (bearing 2 linkers), **5a** (bromo), and **8a** (guanidinium) respectively.

These observations suggested that chemical modification of these peptide mimics affected their hydrogel formation capability. Although reported to increase the antibacterial activity of several peptide mimics^26,33^, the presence hydrophobic bromo substituent might interfere with the π–π stacking interaction of the aromatic group^5^. In addition, the choice of cationic group significantly influenced the balance between hydrophobicity and hydrophilicity of the overall molecules. Changing the cationic group from primary ammonium in hydrogel **2** (Log P = 2.7) to guanidinium in hydrogel **8** (Log P = 1.7) significantly increased the hydrophilicity of the peptide mimics, which may explain their unstable characteristics observed in vial inversion test. Due to their poor structural stability, further characterization of hydrogel **1**, **5**, and **8** was not carried out.

The minimum gel concentration (MGC), the minimum concentration of peptide mimics required to form a self-supporting hydrogel, was qualitatively determined by varying the concentration of peptide mimics **2a**–**4a**, **6a, 7a**, **9a**, and **10a** in a vial inversion test^34–36^. These peptide mimics were found to form self-supporting hydrogels at low concentrations, with MGC ranging from 0.1 to 0.3%(w/v) (Table 1). Additionally, their MGC did not seem to be affected by chemical modifications.

**Table 1:** List of self-supporting NaA-capped cationic hydrogels, their hydrogelation conditions, minimum gel concentration (MGC), and final pH.

Hydrogel	Peptide	Triggers	MGC (%w/v)	pH
1° ammonium **2**	**2a**	GdL, Heat, and NaCl	0.1	5.5
3° ammonium **3**	**3a**	GdL, Heat, and NaCl	0.3	5.5
4° ammonium **4**	**4a**	GdL, Heat, and NaCl	0.2	6
Fluoro **6**	**6a**	GdL, Heat, and NaCl	0.1	5.5
Chloro **7**	**7a**	GdL, Heat, and NaCl	0.2	5.5
TFA **9**	**9a**	Heat and NaCl	0.3	4
Cl **10**	**10a**	Heat and NaCl	0.3	5

### Structural and morphological characterization of hydrogels

#### ^1^H NMR analysis

**Figure 4:**
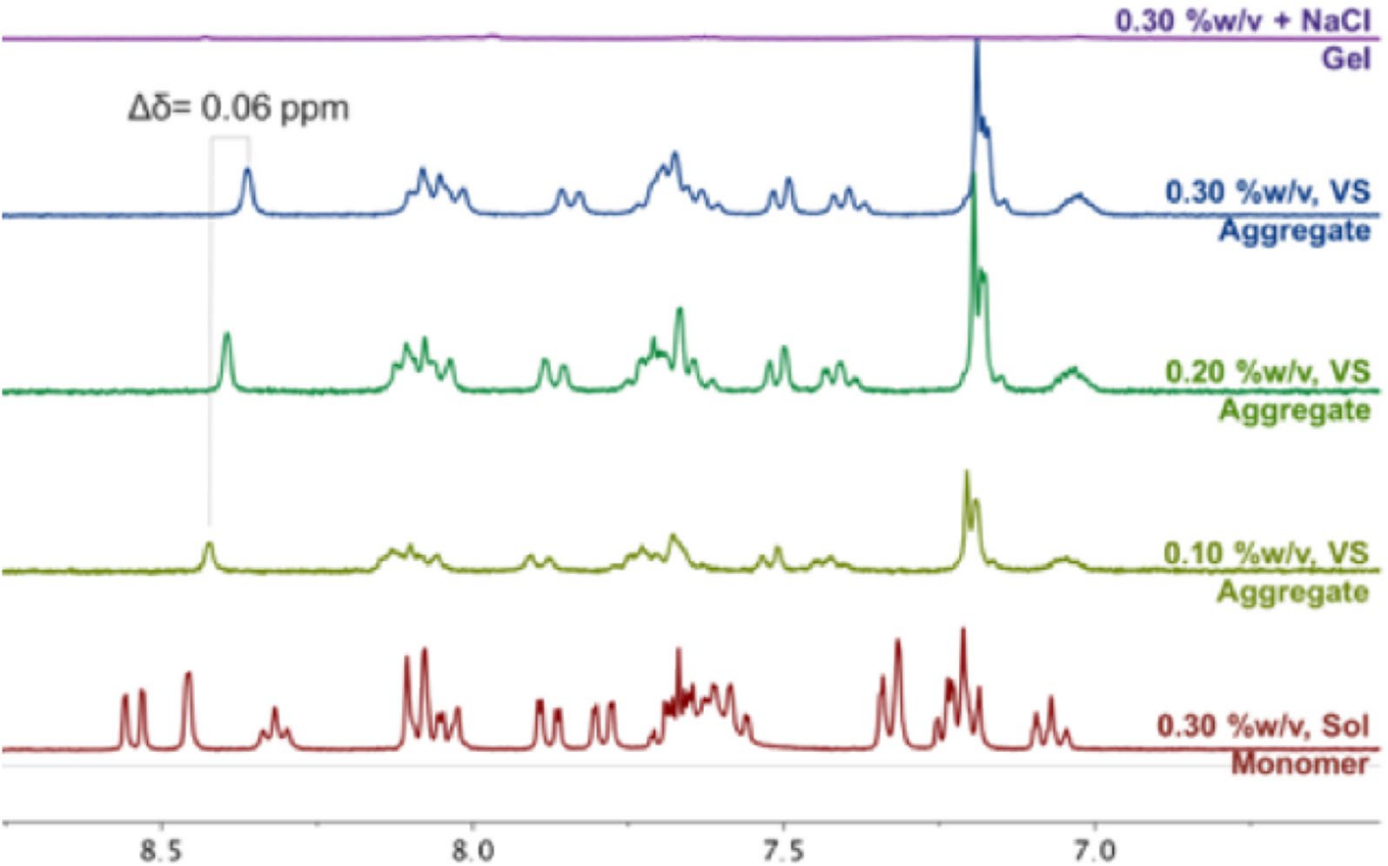
^1^H NMR spectra of NaA-capped cationic peptide mimics **2a** in their monomer, viscous solution (VS), and gel state (hydrogel **2**).

In agreement with the visual observation obtained from vial inversion test, ^1^H NMR spectra also indicated that self-assembly had occurred before the addition of NaCl. The ^1^H NMR of peptide mimic **2a** as a model compound, disolved in DMSO with GDL without heating (monomeric phase), exhibited well-resolved NMR features (Fig. 4, red). Conversely, at the same concentration (0.3%(w/v)) the ^1^H NMR recorded from viscous solution in water showed notably broader peaks (Fig. 4, blue), indicating self-assembly had occured. Aggregates, including fibrils, frequently exhibit broadening of spectral features due to slower tumbling rates on the NMR time-scale^37^.

In addition, the NaA capping group was involved in the self-assembly of these cationic peptide mimics. Upon increasing the concentration from 0.1 to 0.3 %(w/v), an up-field chemical shift (Δδ = 0.06 ppm) of peaks that correspond to the aryl protons of the aromatic-capping group was observed. This indicates their participation in stacking interactions which drive the self-assembly (Fig. 4, green and blue)^25,38–40^.

Addition of NaCl was crucial to drive the equilibrium towards the self-assembled state, leading to self-supporting hydrogel formation. The ^1^H NMR spectrum of hydrogel **2** at 0.3%(w/v) with the addition of NaCl, showed extreme broadening and loss of ^1^H NMR features that indicated complete transformation to their gel phase (Fig. 4, purple)^39^. At low ionic strength, the electrostatic repulsion between the charged molecules might create a barrier to limit self-assembly. Ionic solutes, from the salt, could screen the charges and mitigate the electrostatic repulsion to an extent that hydrogels could be formed^41^.

#### Secondary structure of hydrogel

The secondary structure of the self-assembled hydrogels was investigated by comparing the circular dichroism (CD) and ATR-FTIR spectra of the resulting hydrogels with the spectra of the corresponding peptide mimics. The result demonstrated that structural modification had only showed a subtle change to the CD ellipticity without affecting the over-all secondary structure of the resulting hydrogels (Fig. S2). The CD spectra obtained from hydrogels **2**–**4**, **6, 7**, **9**, and **10** all exhibited the presence of a negative minimum at around 200 nm along with a weak positive maximum around 220 nm, suggesting the formation of a random/disordered coil^42,43^.

Likewise, ATR-FTIR spectra also supported the formation of random/disordered coil within the cationic hydrogels. Both D_2_O gels and xerogels (air-dried gels) made from peptide mimics **2a**–**4a**, **6a, 7a**, **9a**, and **10a** peaks at 1645 ± 4.0 cm^−1^ and 1648 ± 2.0 cm^−1^ which conform to disordered/random coil secondary structures (Table S1)^44^.

#### Morphology of 3-D network of hydrogels

**Figure 5:**
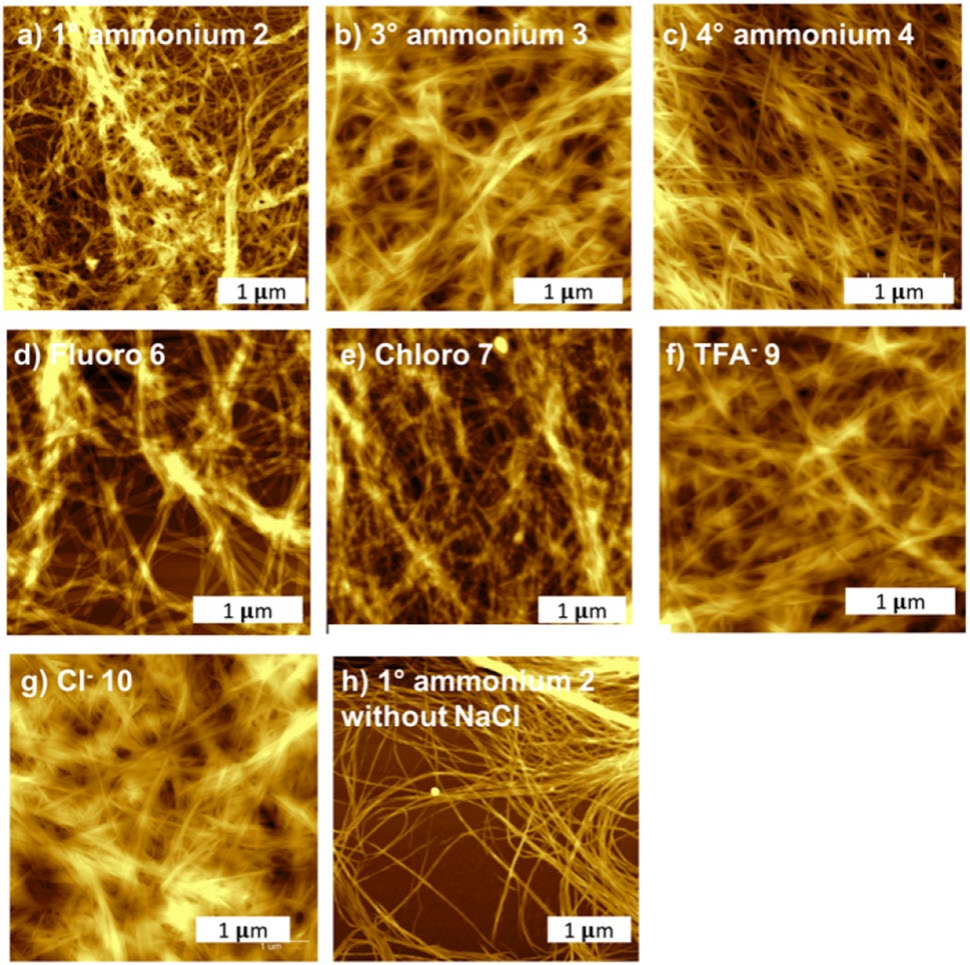
3-D fibrous network observed from hydrogel made from (**a**) primary ammonium **2a**, (**b**) tertiary ammonium **3a**, (**c**) quaternary ammonium **4a**, (**d**) fluoro **6a**, (**e**) chloro **7a**, (**f**) trifluoroacetate **9a**, and (**g**) chloride **10a** which were imaged at 4 × below their MGC. Meanwhile, (**h**) viscous solution of **2a** at 0.05%w/v, without the addition of NaCl, demonstrated fibers with lack of junction zones.

The supramolecular morphologies of hydrogels made from NaA-capped peptide mimics were investigated using AFM. All the xerogels made from peptide mimics **2a**–**4a**, **6a, 7a**, **9a**, and **10a** exhibited nano fibrous network with a lot of junction zones which provided space responsible for immobilizing water molecules leading to hydrogel formation (Fig. 5).

Chemical modifications resulted in variation of the fibers size (diameter) but did not dramatically alter the morphology of the resulting hydrogels (Table 2). Changes on the fiber diameter could affect the mechanical properties of the resulting hydrogel. For instance, thicker fibers, hence smaller pores, resulted in restricted molecule displacement which often correspond with formation of more robust hydrogel^45^.

**Table 2:** NaA-capped cationic hydrogels exhibited fibers with various diameter depending on their chemical structures.

Hydrogel	⌀ (nm)
1° ammonium **2**	115 ± 5
3° ammonium **3**	139 ± 10
4° ammonium **4**	148 ± 16
Fluoro **6**	115 ± 10
Chloro **7**	99 ± 6
TFA^−^ **9**	104 ± 4
Cl^−^ **10**	87 ± 6

Additionally, AFM was also employed to further confirmed that the self-assembly had occurred in the viscous solution without addition of salt. In agreement with the ^1^H NMR and visual observation, fibers (74 ± 6 nm) lacking junction zones or bundling were observed from the viscous solution of peptide mimics **2a** (Fig. 5h). This observation signified that the addition of salt to the viscous solution of peptide mimics **2a** promotes fiber aggregation, which is pivotal in forming a self-supporting hydrogel.

### Mechanical properties

For topical antibacterial application, AMPs have been mixed with hypromellose (a gel base), to obtain a more viscoelastic formulation^46,47^. The self-assembled properties of NaA-capped cationic peptide mimics allow for the formation of viscoelastic hydrogels without the presence of any additive. Frequency sweep test (FST) was performed using a rheometer to precisely examine the viscoelastic properties of the resulting hydrogels. From FST, all of the NaA-capped cationic peptide mimics showed viscoelastic properties consistent with the formation of stable hydrogels. As shown in Fig. S3, the G′ values of hydrogels made from peptide mimics **2a**–**4a**, **6a**, **7a**, **9a**, and **10a** were at least a magnitude higher than their modulus loss (G″), which are indicative of elastic hydrogel characteristic rather than viscous materials^48,49^. In addition, their modulus storage (G′) were also frequency independent, which confirmed the structural stability of these hydrogels, and were comparable with other reported peptide-based self-assembled therapeutic hydrogels^49–52^.

Additionally, the mechanical rigidity of these hydrogels can be modulated by carefully altering the molecular design of the hydrogelators, particularly through variations of cationic group, substituent on the benzene ring of NaA capping group, and counter anion. G′ values often associated with mechanical rigidity of a hydrogel where higher G′ value ascribed to more robust hydrogels^48^. Changing the cationic group of the hydrogel from primary ammonium **2** to tertiary ammonium **3** and quaternary ammonium **4** significantly increase the G′ values from 0.83 to 8.63 kPa and 2.28 kPa, respectively (Table 3). The presence of a methyl group in hydrogels **3** and **4** was thought to decrease their flexibility, which presumably leads to formation of more robust hydrogels^53^.

**Table 3:** Modulus storage (G′) and modulus loss (G″) of hydrogels made from NaA-capped cationic peptide mimics suggested formation of viscoelastic materials.

Hydrogel	G′ (kPa)	G″ (kPa)
1° ammonium **2**	0.83	0.07
3° ammonium **3**	8.63	0.55
4° ammonium **4**	2.28	0.21
Fluoro **6**	1.20	0.06
Chloro **7**	0.53	0.50
TFA^−^ **9**	5.12	0.31
Cl^−^ **10**	1.61	0.16

Moreover, the mechanical rigidity gradually decreased as larger electron withdrawing group (EWG) was introduced on the NaA-capping group. The presence of a relatively small EWG, i.e. fluoro in hydrogel **6**, resulted in a decrease of G′ value from 8.63 to 1.20 kPa (Table 3). A further decrease in G′ value (0.53 kPa) was observed for hydrogel **7**, bearing a chloro substituent. Nevertheless, both fluoro **6** and chloro **7** demonstrated prominent characteristics of stable hydrogels (Fig. S3). This observation suggested that the electronegative and steric effects of substituents can be used to fine-tune the mechanical properties of the resulting hydrogels^54^.

Furthermore, changing the anion from gluconate (hydrogel **3**) to trifluoroacetate (hydrogel **9**) resulted in a subtle change in the rigidity of the hydrogel. However, the hydrogel **10** (bearing a chloride anion) exhibited a notable decrease of G′ value, presumably due to the absence of H-bonding capability of the counter anion (Cl^−^)^55^.

### Antibacterial activity of the ultrashort cationic hydrogels

*Staphylococcus aureus* (*S. aureus*) is one of the major causative bacteria for skin and soft tissue infections^56,57^. Therefore, the NaA-capped cationic hydrogels were challenged against an inoculation of 3 × 10^4^ CFUs/mL of *S. aureus* using a modification of a previously reported method^58^.

**Figure 6:**
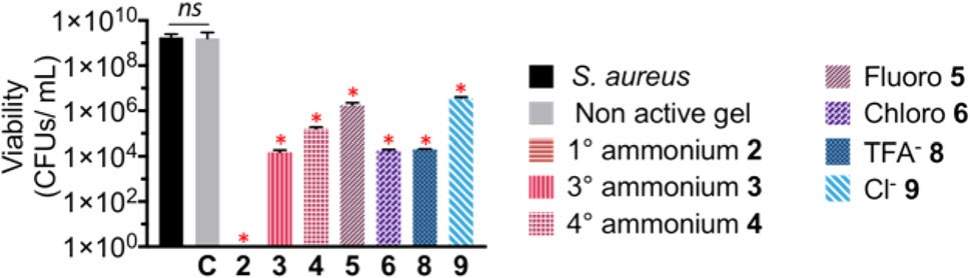
Antibacterial activity of NaA-capped cationic hydrogel **2**–**4**, **5**–**6**, and **8**–**9** showed significant bacterial reductions ranging from 3.0 Log_10_ and > 9.0 Log_10_ against *S. aureus*. n = 3; *p* < 0.0001.

Hydrogel **2,** bearing primary ammonium group, was found to be the most active and exhibited complete killing against *S. aureus* with a reduction of > 9.0 Log_10_ CFUs/mL compared to the controls after 18 h growth (Fig. 6). This hydrogel demonstrated improved activity when compared to previously reported short peptide-based hydrogels in combating *S. aureus*^58–61^. Additionally, hydrogel **2** exhibited significant bacteria reduction (5.5 Log_10_) even when it was tested against a higher inoculum of 10^8^ CFUs/mL *S. aureus* colonies (Fig. S4).

The architecture of the cationic groups plays a significant role in determining the antibacterial activity in solution. For example, anthranilamide-derived peptide mimics bearing cationic charges have been reported to exhibit a range of antibacterial properties against Gram-positive (MIC 2.0–62.5 µM) and Gram negative (MIC 15.6–125 µM) bacteria^26^. Similarly, hydrogel having the tertiary ammonium **3a** and quatenary ammonium **4a**, showed 5.1 Log_10_ and 4.1 Log_10_ reductions, respectively. The presence of methyl substituent on the cationic moiety seems to reduce the antibacterial activity of the resulting hydrogels, presumably due to the decrease in membrane binding capability^62,63^.

In contrast to the finding by Kuppusamy et al.^26^, the presence of a halogen atom and variation of the counter anion did not dramatically alter the antibacterial properties of the parent hydrogel **3**. The hydrogels bearing fluoro (**6**), chloro (**7**), TFA^−^ (**9**), and Cl^−^ (**10**) exhibited 3.1 Log_10_, 5.0 Log_10_, 4.9 Log_10_, and 3.0 Log_10_, respectively.

The rheological properties, specifically the storage modulus G′, was reported to play a significant role in determining the antibacterial properties of multi domain peptide (MDP) hydrogels by providing a mechanical support to the fibrous networks against the restrained bacteria^64^. Surface chemistry of the supramolecular hydrogel nanofibers and their bulk rheological properties were thought to have a large effect on its antimicrobial activity. In contrast, no trend could be observed between the G′ value of the gels and their antibacterial activity in this study. This result suggests that the NaA-capped hydrogel inhibites bacteria growth in a different mechanism to MDP hydrogel.

To widen the scope of antibacterial study, the most active hydrogel against *S. aureus* made from primary ammonium **2a** (hydrogel **2**) was challenged against *E. coli*, a Gram-negative bacteria which is also associated with skin and soft tissue infection (SSTI)^65–67^. Hydrogel **2** showed notable antibacterial activity (6.4 Log_10_ reduction) against *E. coli* (Fig. S5). The difference in antibacterial activity between Gram-positive and Gram-negative was presumably due to the presence of additional outer bilayer membrane consist of lipopolysaccharide and phospholipid in Gram-negative bacteria^68^.

### Antibacterial activity of the fibers released from hydrogel 2

Mechanism of antibacterial actions for most short peptide-based hydrogels is still not fully understood, however one plausible mechanism is the release of active substance from the parent hydrogel. To examine this hypothesis, hydrogel **2** were immersed in water over 10 days and the release of the substance was monitored using UV/Vis spectroscopy.

**Figure 7:**
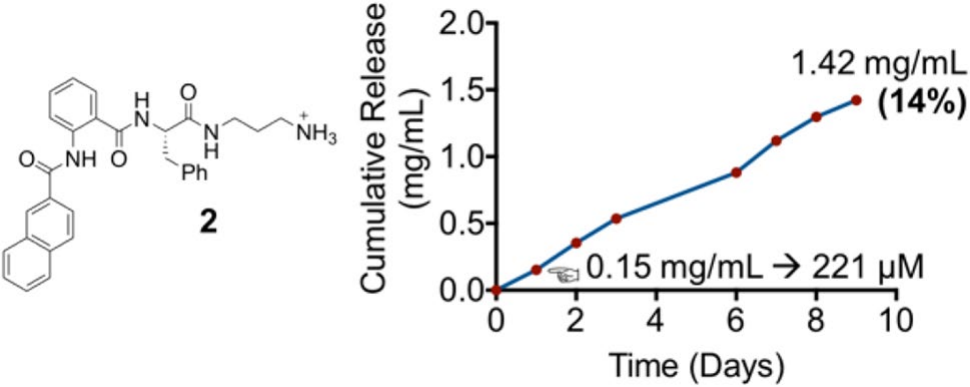
Hydrogel **2** at 1%w/v exhibited a sustainable released profile over 9 days. The graph represents two individual measurement and the some of the error bars are on the order of the graph point size.

The UV/Vis spectra showed peaks which centered at 235 nm and 290 nm. This result suggested that the cationic peptide mimics was released from hydrogel **2** and could account for the antibacterial activity observed. After incubation at 37 °C for 24 h, 0.15 mg/mL (221 µM) was quantified to be released from primary ammonium hydrogel **2**. Furthermore, a continuous release profile was observed over 9 days with ~ 14% of total peptide mimics was released at the end of experiment (Fig. 7).

Additionally, hydrogel **2** was subjected to a disk diffusion assay. A zone of inhibition with diameter of 2.3 cm was observed. This finding further demonstrates that an active agent was released from hydrogel **2**.

**Figure 8:**
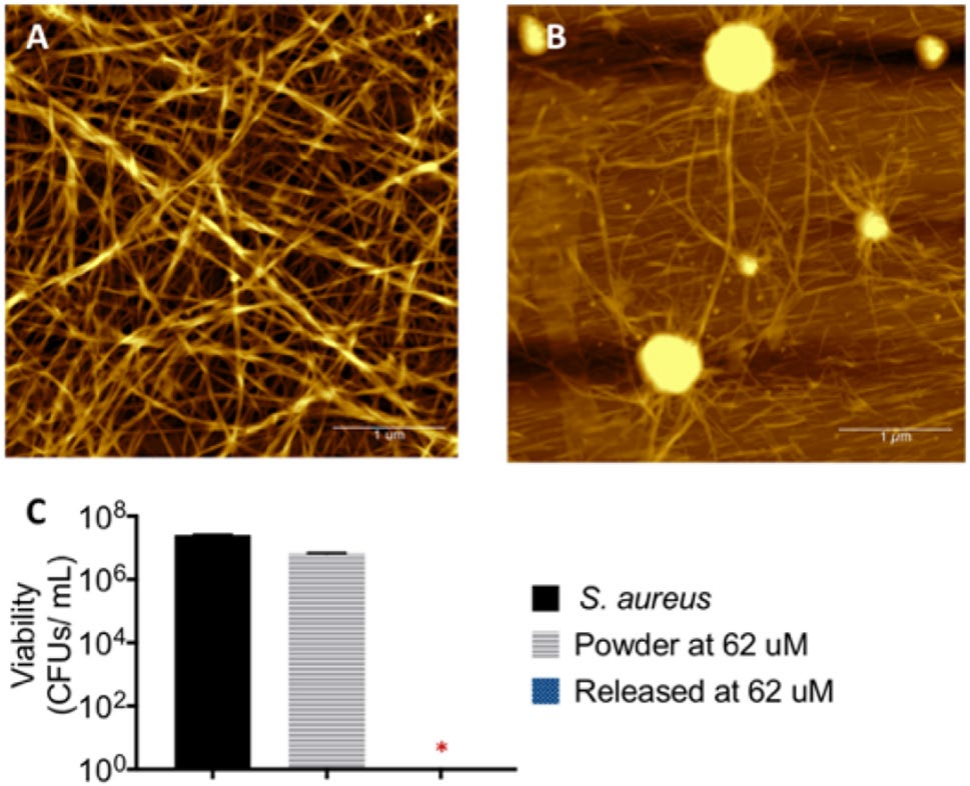
(**a**) Fibers with diameter of 47 ± 4.9 nm were observed from released solution of hydrogel **2**; (**b**) AFM images of cationic peptide mimics **2a** at 221 µM, which was prepared with DMSO (5%): water without forming the hydrogel, showed a mixture of thin fibers with lack of junction zones and spheroidal aggregates. Scale bar of AFM images was denoted as 1 µm. and (**c**) fibers released solution from hydrogel **2** exhibited bactericidal activity, with complete kill observed against *S. aureus*, meanwhile peptide mimics **2a** as a monomer did not show notable reduction.

AFM was performed on the supernatant solution to examine whether the supernatant solution was composed of monomers or intact nanofibers. Supramolecular nano fibers with diameter of 47 ± 4.9 nm were observed (Fig. 8a) despite the concentration of the peptide mimics at below their MGC (1 mg/mL) in the supernatant.

The antibacterial efficacy of the released nano fibers collected from supernatant solutions on day 1–9 were evaluated using a microdilution protocol^69^. The nano fibers were found to have a minimum inhibitory concentration (MIC) of 62 µM (Fig. 8c) against *S. aureus*. As seen in Fig. 7, the hydrogel released nanofibers above the active concentration within all 24 h periods across the 9 days.

Supramolecular nano fibers have been reported to enhance the antibacterial activity, presumably due to higher local cationic densities on the fibrils^50^. Interestingly, the antibacterial activity of NaA-based cationic peptide mimics also relies on the self-assembled morphologies. At the same concentration (62 µM), cationic peptide mimics **2a** (as a free compound) did not showed significant bacteria reductions (< 1Log_10_ CFU/mL) (Fig. 8c, grey). AFM images obtained for short cationic peptide mimics **2a**, which was prepared without forming the hydrogel, showed a mixture of fibers with spheroidal aggregates and lack of junction zones (Fig. 8b).

### Cytotoxicity of hydrogel 2

Aside from exhibiting antibacterial properties, ideal candidates for antibacterial biomaterial need to exhibit low toxicity against mammalian cells. Therefore, the cytotoxicity of hydrogel **2** made from primary ammonium **2a**, at various concentrations was examined against HEK293T cells.

**Figure 9:**
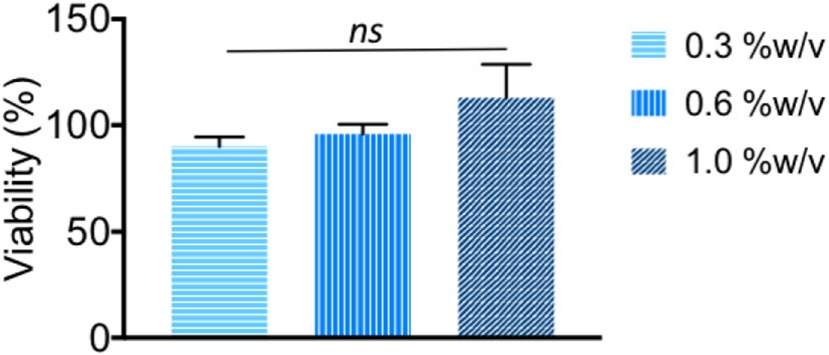
Hydrogel **2** exhibited low toxicity against HEK293T cells for all concentrations tested, suggesting their low toxicity. *ns*: not significant.

More than 80% cells viability were observed indicating that hydrogel **2** was not toxic to normal cells (Fig. 9). The remarkable antibacterial activity and low toxicity of hydrogel **2** makes it a promising candidate for development as an antibacterial biomaterial.

## Conclusions

A library of ultra-short NaA-capped hydrogelators having only one amino acid (l-phenylalanine) have been synthesised in excellent yields. Majority of the synthesised cationic peptide mimics efficiently formed hydrogels at low concentration under physiological pH. The resulting hydrogels adopted disordered coil as secondary structures and exhibited desirable mechanical properties, with rigidity modulated by either varying the cationic group, substituent on the anthranilamide-core, or counter anion. The peptide mimics are derived simply from readily available starting materials and the reactions are done in solution phase, which are more amenable to large-scale synthesis compared to current peptide based LMWG. We have also demonstrated the structural flexibility and versatile control over the many structural variations that can be constructed in NaA-capped gelators. This class of gelators represent a substantial departure from other LMWGs, enabling, for the first time, a systematic examination of the effects of different chemical moieties on intramolecular interactions which induce self-assembly and hydrogel formation, while also enabling function such as antibacterial activity.

The NaA-capped cationic hydrogels, particularly primary ammonium **2**, displayed promising antibacterial properties with significant bacteria reduction against *S. aureus* (> 9.0 Log_10_) and *E. coli* (6.4 Log_10_). Furthermore, the antibacterial activities of these hydrogels were attributed to the released of nanofibers. The most active hydrogel primary ammonium **2** demonstrated a continuous nanofiber released profile that was antibacterial over 9 days. Additionally, this hydrogel also exhibited low toxicity (> 80% viability) against HEK293T cells.

These intrinsically antibacterial and non-toxic hydrogels are ideal candidates for further development in applications where bacterial contamination is problematic.

## Experimental

All chemicals and solvents used were purchased from Chemimpex, Combi Blocks, and Sigma Aldrich and were used directly without any further purification.

### Synthesis

The Naphthyl anthranilamide-capped cationic peptide mimics **1a**–**10a** were synthesized via ring-opening reaction of isatoic anhydride derivatives as previously reported^25^. The scheme and synthetic procedures to obtain hydrogelators **1**–**10** along with their characterization data (^1^H NMR, ^13^C NMR, and HRMS) are given in the supplementary information.

### Preparation of hydrogels

Respective amount of hydrogelator 1–10 was added to each vial with internal diameter (i.d.) of 10 mm containing Mili-Q water. For, hydrogelators 1–8, GdL (1.5 equivalents) was subsequently added. The resulting suspensions of 1–10 were slowly heated until all of the solid completely dissolved. Afterwards, NaCl (5 equivalents) was added to each vial to make up total volume of 1 mL, followed by gentle mixing. The resulting clear solution was left at room temperature without disturbance for 3 h. The vials were then inverted to confirm the hydrogel formation. MGC, represent as a percentage, is the minimum weight of hydrogelator required to form a self-supporting hydrogel divided by the total volume.

### ^1^H NMR spectroscopy

The NMR sample of the monomer was prepared by dissolving 2 mg of primary ammonium **2a** in 650 µL of DMSO d-_*6*_. Meanwhile, the NMR sample of the viscous solution was prepared by dissolving respective amount of **2a** and GdL in 650 µL of D_2_O to make up concentration of 0.1, 0.2, and 0.3%w/v. Subsequently, NaCl was added to the viscous solution of **2a** at 0.3%w/v and the spectra was recorded once again using a Bruker Avance III 400 MHz NMR spectrometer. NMR spectra were processed using MestreNova software.

### Circular dichroism spectroscopy

Hydrogels **2**–**4**, **6**, **7**, **9**, and **10** were prepared at 1%w/v and were diluted 8 times in Mili-Q water before being transferred into a 0.2 mm path length cuvette. The spectra were collected using ChirascanPlus CD spectrometer (Applied Photophysic UK) scanning wavelengths of 180–500 nm with a bandwidth of 1 nm, 0.6 s per point, and step of 1 nm. Each experiment was performed in triplicate and the results were averaged into a single plot value.

### Attenuated total reflectance infrared spectroscopy

The ATR-FTIR spectra were obtained using a Spectrum 100 FTIR spectrometer (PerkinElmer, USA) fitted with a 1 mm diamond-ZnSe crystal. Xerogels were made in situ by applying nitrogen flow to one drop of the pre-formed hydrogels (2%w/v) which was place on the ATR-crystal. The spectra of xerogels **2**–**4**, **6**, **7**, **9**, and **10** were recorded from 4000 to 650 cm^−1^ with a 4 cm^−1^ resolution and 4 scans.

The spectra of D_2_O gels were measured by applying two drops of pre-formed gels at 2%w/v on the ATR crystal which was then recorded from 4000 to 650 cm^−1^ with a 4 cm^−1^ resolution and 4 scans.

### Atomic force microscopy (AFM)

Hydrogels 2–**4**, **6**, **7**, **9**, and **10** were prepared from the corresponding cationic peptide mimics at their MGC, 2 × below, and 4 × below MGC in glass vials. Prior to gelation, one drop of these peptide mimics solutions was casted onto a mica substrate. Using a glass slide, each droplet was carefully spread and was left to dry overnight before imaging. Imaging was performed using a Bruker Multimode 8 Atomic Force Microscope in Scanasyst Air (PeakForce Tappings) mode, which is based on tapping mode AFM. To prevent damage of soft samples, the imaging parameters were constantly optimized through the force curves that were collected. Bruker Scanasyst-Air probes were used, with a spring constant of 0.4–0.8 N m^−1^ and a tip radius of 2 nm.

To gain some insight on the mechanism of antibacterial action, the released solutions from hydrogel **2** was directly casted on to a mica substrate. In addition, respective amount peptide mimics **2a** and GdL were dissolved in DMSO-Water (5%: 95%) which then diluted to obtained concentration of 221 µM before casted on a mica substrate. After gently spread using a glass slide and left to dried overnight, these samples were imaged using a similar manner as describe above.

### Rheology measurement

The mechanical properties of NaA-capped cationic hydrogels were assessed using an Anton Paar MCR 302 rheometer with a 25 mm stainless parallel plate configuration.

Initially, 1 mL of hydrogels were prepared from peptide mimics **2a**–**4a**, **6a**, **7a**, **9a**, and **10a** in glass vials at concentration of 1%w/v. These vials were warmed using a heat gun to transform the hydrogel to the solution phase. Subsequently, 560 µL of the resulting solutions were cast onto the rheometer plate. The other plate was lowered to the measuring position (1 nm) and the hydrogel was allowed to stand for 3 h for the gel to form. Initially strain sweep test (SST) was performed at a frequency of 1 Hz using 0.1% strain to 100% strain to identified the linear viscoelastic region of each hydrogel. Subsequently, frequency sweep test (FST) was conducted at fixed strain of 0.1%, which is within the LVER of all hydrogels, and frequency ranging from 10 to 0.01 Hz. The rheology data were shown as the average of three repeats for each data point.

### In vitro released of fibers from hydrogel 2

Hydrogel **2** was made at 1%w/v with final volume of 1 mL using the same method as described above. The resulting hydrogel was left to stand overnight at room temperature. PBS (1 mL) was added gently from the side wall to avoid physical fractures. Subsequently, the vial was incubated at 37 °C, where 1 mL of PBS was sampled at each time point and replaced with fresh PBS. The samples were subjected to 10 × dilution before quantified using UV–Vis spectroscopy. This experiment was performed in triplicate.

### Antibacterial assay

A single colony of *S. aureus* 38 or *E. coli K12* was grown overnight in Laura Brentani (LB) broth at 37 °C. The resulting suspension were centrifuged and re-suspended in the same volume of LB twice. The optical density (OD) of the resulting bacteria culture was adjusted to 0.1 at 600 nm in LB (10^8^ CFUs/mL) which further adjusted to 3 × 10^4^ CFUs/mL. Bacteria solutions (1 mL), which contain either 10^8^ CFUs/mL or 3 × 10^4^ CFUs/mL, was gently added to hydrogel **2** at 1%w/v with total volume of 1 mL. After being incubated at 37 °C for 18 h, 100 µL of bacteria solution in each vial was taken and subjected to serial dilution using phosphate buffer solution (PBS). 20 µL of each dilution were carefully transferred into nutrient agar plates and incubated for another 18 h. The following day, bacterial growth inhibition was quantified using the viable count method. The experiment was performed twice in triplicate and multiple sample comparison was performed using one-way ANOVA.

To further investigate their antibacterial activity, the released fibers from hydrogel **2** were tested against *S. aureus 38*. The solution taken from day 1 (contain ~ 221 uM) was adjusted to obtained final concentrations of 125, 62.5, 31.25, and 18 µM. On the other hand, monomers solution of cationic peptide mimics **2a**, that has not formed hydrogels, was prepared by dissolving a known amount of peptide mimics **2a** in DMSO to give a 20 mM stock solution which then diluted with LB to make up concentration of 125, 62.5, 31.25, and 18 µM. 100 µL of the above peptide mimics monomer and fiber solution was transferred to a 96 well plates.

Subsequently, 100 µL bacteria solution (10^6^ CFUs/mL) was added to each well containing samples. A blank control was used: one containing 100 µL of bacteria solution and 100 µL of PBS. The plate was then incubated at 37 °C for 24 h. The following day, 20 µL solution from each well was subjected to a serial dilution then transferred onto agar plates followed by incubation at 37 °C for another 24 h. MIC value was determined as the lowest concentration that inhibited *S. aureus* growth on the agar plate^70^.

### Cytotoxicity assays

Cytotoxicity measurements were performed in HEK293T cells using an alamarBlue colorimetric assay. Each experiment was repeated at least three times^25^. Cells were passaged using standard cell culture procedures. Cells were detached with trypsin and centrifuged (1000 rpm for 3 min). The supernatant was removed and the cells re-suspended in Dulbecco’s Modified Eagle Medium (DMEM) at a concentration of 100,000 cells per mL. Cells were seeded at a concentration of 6,000 cells per well. For cytotoxicity measurements, 100 mL of hydrogel **2** at 0.3, 0.6, and 1.0%w/v were added in triplicate to a 96-well plate and allowed to set overnight. Surrounding wells were supplemented with water to ensure hydration of the gels. Gels were then incubated for 24 h with DMEM. Cells were seeded atop the hydrogels and incubated for 24 h. 10 mL of Alamar Blue was added to the wells, followed by further incubation for 4 h. Control wells included cell-free gels, no hydrogels and a negative control of 15% (v/v) DMSO. The absorbances at 570 nm and 596 nm were recorded using a BioRad Benchmark plate reader.

## Supplementary Information

Supplementary Information.

## Data Availability

The datasets used and/or analysed during the current study are available from the corresponding author on reasonable request.
