# Supplementary material for: Hydrogels with intrinsic antibacterial activity prepared from naphthyl anthranilamide (NaA) capped peptide mimics
Source: Sci Rep. 2022 Dec 23;12:22259. doi: 10.1038/s41598-022-26426-1 (PMC9789043; doi:10.1038/s41598-022-26426-1)
Supplement: Supplementary file 1 — Supplementary Information. [file 41598_2022_26426_MOESM1_ESM.docx]

Supporting Information

Hydrogels with Intrinsic Antibacterial Activity Prepared from Naphthyl Anthranilamide (NaA) Capped Peptide Mimics

Vina R. Aldilla, Renxun Chen^*^, Rajesh Kuppusamy, Sudip Chakraborty, Mark D. P. Willcox, David StC. Black, Pall Thordarson, Adam D. Martin, Naresh Kumar^*^

**Figure S 1** (a) Viscous solution of naphthyl anthranilamide (NaA) capped cationic peptide mimics. (b) Addition of salt turned the viscous solution to self-supporting hydrogel (left), however for peptide mimics **1a**, **5a** and **8a** water was elapsed from these hydrogels followed by structural disintegration (right)

**Figure S 2** Circular dichroism (CD) spectra of hydrogels made from NaA-capped short cationic peptide mimics bearing (a) different cationic groups **2a**-**3a**, (b) halogen as a substituent **6a**-**7a**, and (c) various counter anion suggested random/ disordered coil as their secondary structure.

**Figure S 3** Frequency sweep test (FST) performed using a rheometer on hydrogels made from (a) primary ammonium **2a**, (b) tertiary ammonium **3a**, (c) quaternary ammonium **4a**, (d) fluoro **6a**, (e) chloro **7a**, (f) TFA **9a**, and (g) Cl **10a** at 1 %w/v showed formation of stable hydrogel as indicated by their significantly higher modulus storage (G´) value compared to their modulus loss (G´´).

**Figure S 4** Hydrogel **2** exhibited 5.5. Log_10_ reduction, although challenged against higher *S. aureus* inoculums (10^8^ CFU/mL). n= 3; p< 0.0001

**Figure S 5** Hydrogel **2** showed notable bacteria reduction (6.2 Log_10_) against *E. coli*. Meanwhile, the non-active hydrogel which used as a control did not show significant bacteria reduction. n= 3, p <0.0001

**Table S 1** ATR-FTIR spectra of NaA-capped ultra-short cationic peptide showed peaks corresponding to random coil secondary structures.

**Scheme S 1** Synthesis scheme to obtain hydrogelators **1**-**10**

**Scheme S 2** Addition of glucono-δ-lactone (GdL) to primary amines **2a** provided primary ammonium gluconate salts leading to dissolution of this compound upon heating.

Synthesis

General Procedures (GP)

General procedure for ring-opening isatoic anhydride derivatives (**GP1**)

As previously reported, L-phenylalanyl hydrochloride salt (F•HCL) (1.0 equivalent) was dissolved in Milli-Q water. Potassium carbonate (2.0 equivalents) was added and the reaction mixture was stirred at room temperature for 20 minutes. After the reaction mixture turned clear, isatoic anhydride derivatives (1.0 equivalent), dissolved in acetone, were added and allowed to react at room temperature for another 18 hours. After completion, the reaction mixture was removed under reduced pressure to remove the acetone. The resulting precipitate was then filtered and dried to provide intermediates **11a-d** as white solids in 65-73% yield.

General procedure of naphthoylation of compounds **12a-d** (**GP2**)

The ring-opened isatoic anhydride derivatives **11a-d** were suspended in anhydrous DCM (1-5 mL) under argon atmosphere, followed by addition of Et_3_N (2.0 equivalents) and DMAP (0.1 equivalents). After stirring at room temperature for 10 minutes, the reaction mixture was cooled down to 0 °C and naphthoyl chloride (1.5 equivalents) was added slowly. Afterwards, the reaction mixture was warmed to room temperature and stirred for 4-6 hours. After completion, the reaction mixture was removed under reduced pressure. The resulting crude material was purified using column chromatography with hexane: ethyl acetate to afford **12a-d** as white solids in 80-90% yield.

General procedure for hydrolysis (**GP3**)

The methyl ester-protected intermediates **12a-d** were dissolved in THF: MeOH: H_2_O with volume ratio of 10:5:2. Lithium hydroxide (LiOH) (3.0 equivalents) was added to the reaction mixture which was then stirred for 18 hours. The reaction mixture was diluted with Milli-Q water (20 mL) and washed with diethyl ether (3x15 mL). The aqueous phase was acidified until pH 3-4 and was extracted with ethyl acetate (2x25 mL). The resulting organic phase was dried over sodium sulfate and concentrated under reduced pressure to afford **13a-d** as white solids in quantitative yield.

General procedure of amide coupling (**GP4**)

Compounds **13a-d**, bearing a carboxylic acid end (1.0 equivalent), were dissolved in anhydrous DMF under nitrogen atmosphere followed by addition of HOBT (1.2 equivalents). The reaction mixture was cooled down to 0 °C and stirred for 30 minutes. Boc-protected diamines or *N*,*N*-dimethylpropane-1,3-diamine (1.1 equivalents) in DMF were then added dropwise to the cooled reaction mixture. Subsequently, EDC•HCl (1.2 equivalents) was added followed by the addition of DIPEA (2.0 equivalents). The cloudy reaction mixture was warmed to room temperature and stirred for 18 hours. After completion, the reaction mixture was poured into ice-water mixture, filtered, and dried. The resulting crude solids were purified by column chromatography to give the pure solids of in 50-78% yields.

General procedure for Boc-deprotection to obtain **5a**, **1a**, and **2a** (**GP 5**)

Boc-protected compounds were dissolved in anhydrous DCM under nitrogen atmosphere. The clear solution was cooled down to 0 °C for 10 minutes followed by addition of either TFA or HCl (4M in dioxane) (2.0 equivalents). The reaction mixture was allowed to warm to room temperature and stirred for 2-18 hours. After completion, indicated by TLC, the reaction mixture was concentrated under reduced pressure to remove excess solvent and acid. The resulting crude material was washed by diethyl ether, filtered, and dried to provide **5a**, **1a**, and **2a** in quantitative yield.

Methyl (2-aminobenzoyl)-L-phenylalaninate **11a**[1]

According to **GP1**, L-Phenylalanine methyl ester hydrochloride (F•HCl) (4.0 g, 19.0 mmol) was used to ring open isatoic anhydride (1.0 equivalent) to give compound **11a** as a white pure solid (3.7 g, 65% yield).

^1^H NMR (400 MHz, DMSO- *d*_6_) 8.62 (1H, d, *J* = 7.6 Hz, *NH*), 7.56 (1H, d, *J* = 7.8 Hz *ArH*), 7.36–7.41 (4H, m, *ArH*), 7.28–7.31 (1H, m, *ArH*), 7.21–7.25 (1H, m, *ArH*), 6.76 (1H, d, J= 8.4 Hz, *ArH*), 6.59 (1H, t, J= 7.8 Hz, *ArH*), 6.42 (2H, bs, *NH_2_*), 4.67–4.73 (1H, m, *CH*), 3.73 (3H, s, *OCH_3_*), 3.15–3.26 (2H, m, *CH_2_*).

Methyl (2-amino-4-fluorobenzoyl)-L-phenylalaninate **11b**

F•HCl (1.0 g, 4.6 mmol) was reacted with 5-fluoroisatoic anhydride (1.0 equivalent), as described in **GP1**, to afford **11b** as a pure white solid (1.20 g, 82% yield).

IR: 3463, 3370, 3325, 3022, 2958, 2111, 1745, 1631, 1594, 1511, 1440, 1371, 1296, 1223, 1174, 986, 946, 880, 812, 756, 697. ^1^H NMR (400 MHz, DMSO-*d_6_*) 8.61 (d,1H, *J* = 7.7 Hz, *NH*), 7.37–7.25 (m, 5H, *ArH*), 7.25–7.14 (m, 1H, *ArH*), 7.05 (ddd, 1H, *J* = 9.0, 8.1, 3.0 Hz, *ArH*), 6.68 (dd, 1H, *J* = 9.0, 5.0 Hz, *ArH*), 6.23 (s, 2H, *NH_2_*), 4.60 (ddd, 1H, *J* = 10.1, 7.6, 5.3 Hz, *CH*), 3.63 (s, 3H, *OCH_3_*), 3.19–3.01 (m, 2H, *CH_2_*). ^13^C NMR (101 MHz, DMSO*-d*_6_) 172.24, 167.93, 151.34, 146.58, 137.73, 129.02, 128.25, 126.50, 119.71, 119.48, 117.65, 117.58, 113.75, 113.53, 113.16, 113.10, 53.99, 51.94, 36.11. HRMS (ESI): calcd for C_17_H_17_FN_2_O_3_+Na: 339.1121 found 339.1128.

Methyl (2-amino-4-chlorobenzoyl)-L-phenylalaninate **11c**

F•HCl (1.0 g, 5.1 mmol) was treated with 5-chloroisatoic anhydride (1.0 equivalent) to give **11c** as a white solid (1.48 g, 87% yield).

IR: 3448, 3353, ,3316 3030, 2955, 2658, 2116, 1741, 1632, 1585, 1519, 1441, 1371, 1303, 1258, 1217, 1173, 1107, 981, 818, 758, 699. ^1^H NMR (400 MHz, DMSO-*d*_6_) 8.70 (1H, d, *J* = 7.7 Hz, *NH*), 7.54 (1H, d, *J* = 2.5 Hz, *ArH*), 7.28 (4H, d, *J* = 4.3 Hz, *ArH*), 7.24–7.12 (2H, m, *ArH*), 6.69 (1H, d, *J* = 8.8 Hz, *ArH*), 6.47 (2H, s, *NH*), 4.60 (1H, ddd, *J* = 10.0, 7.7, 5.4 Hz, *CH*), 3.63 (3H, s, *OCH_3_*), 3.20–3.01 (2H, m, *CH_2_*). ^13^C NMR (101 MHz, DMSO*-d*_6_) 172.24, 167.78, 148.71, 137.73, 131.81, 129.01, 128.26, 127.56, 126.51, 118.06, 117.61, 114.30, 53.96, 51.94, 36.09. HRMS (ESI): calcd for C_17_H_17_ClN_2_O_3_+Na: 355.0825 found 355.0820.

Methyl (2-amino-4-bromobenzoyl)-L-phenylalaninate **11d**

According to **GP1**, F•HCl (1.0 g, 4.1 mmol) was reacted with 5-bromoisatoic anhydride (1.0 equivalent) to afford **11d** as an off-white solid (1.3 g, 80% yield).

IR: 3447, 3353, 3313, 3026, 2951, 1741, 1616, 1582, 1438, 1368, 1303, 1259, 1216, 1163, 1097, 979, 816, 757, 698. ^1^H NMR (300 MHz, DMSO-*d*_6_) 8.71 (1H, d, *J* = 7.7 Hz, *NH*), 7.66 (1H, d, *J* = 2.4 Hz, *ArH*), 7.28 (4H, d, *J* = 4.4 Hz, *ArH*), 7.26–7.17 (2H, m, *ArH*), 6.65 (1H, d, *J* = 8.8 Hz, *ArH*), 6.49 (2H, s, *NH_2_*), 4.60 (1H, ddd, *J* = 9.8, 7.6, 5.6 Hz, *CH*), 3.63 (3H, s, *OCH_3_*), 3.21–2.96 (2H, m, *CH_2_*). ^13^C NMR (101 MHz, DMSO*-d*_6_) 172.23, 167.68, 149.02, 137.72, 134.48, 130.38, 129.00, 128.24, 126.50, 118.45, 114.94, 104.78, 53.95, 51.93, 36.09. HRMS (ESI): calcd for C_17_H_17_ClN_2_O_3_+Na: 399.0320 found 399.0327.

Methyl (2-(2-naphthamido)benzoyl)-L-phenylalaninate **12a**

Compound **11a** (3.0 g, 10 mmol) was reacted with naphthoyl chloride (1.5 equivalents) according to **GP2** to provide compound **12a** as a white fluffy solid (4.0 g, 90% yield).

IR: 3364, 2953, 1743, 1676,1598, 1520, 1441, 1368, 1289, 1215, 1117, 1093, 980,914, 858, 756, 716. ^1^H NMR (400 MHz, DMSO-*d*_6_) 12.08 (1H, s, *NH*), 9.28 (1H, d, *J* = 7.9 Hz, *NH*), 8.62 (1H, dd, *J* = 8.4, 1.2 Hz, *ArH*), 8.46 (1H, d, *J* = 1.8 Hz, *ArH*), 8.14–8.06 (2H, m, *ArH*), 8.06–8.00 (1H, m, *ArH*), 7.89 (1H, dd, *J* = 8.6, 1.9 Hz, *ArH*), 7.78 (1H, dd, *J* = 7.9, 1.5 Hz, *ArH*), 7.71–7.63 (2H, m, *ArH*), 7.63–7.57 (1H, m, *ArH*), 7.35–7.27 (2H, m, *ArH*), 7.27–7.19 (3H, m, *ArH*), 7.15–7.06 (1H, m, *ArH*), 4.81 (1H, ddd, *J* = 10.4, 7.8, 5.0 Hz, *CH*), 3.65 (3H, s, *OCH_3_*), 3.28–3.03 (2H, m, *CH_2_*). ^13^C NMR (101 MHz, DMSO*-d*_6_) 172.69, 168.77, 164.45, 139.14, 137.95, 134.43, 132.44, 132.21, 131.81, 129.12, 129.05, 128.69, 128.35, 128.14, 127.77, 127.70, 127.08, 126.38, 123.21, 122.88, 120.34, 120.22, 54.12, 51.90, 36.17. HRMS (ESI): calcd for C_28_H_24_N_2_O_4_+Na: 475.1632 found 475.1626

Methyl (2-(2-naphthamido)-4-fluorobenzoyl)-L-phenylalaninate **12b**

According to **GP2**, compound **11b** (1.0 g, 3.1 mmol) was reacted with naphthoyl chloride (1.5 equivalent) to give **12b** as a pure white fluffy solid (1.2 g, 82% yield). IR: 3328, 3031, 2952, 2108, 1898, 1743, 1671, 1601, 1517, 1445, 1409, 1297, 1251, 1198, 1167, 960, 867, 814, 753, 700. ^1^H NMR (400 MHz, DMSO-*d*_6_) 12.08 (1H, s, *NH*), 9.33 (1H, d, *J* = 7.9 Hz, *NH*), 8.74 (1H, dd, *J* = 9.2, 5.3 Hz, *ArH*), 8.56–8.39 (1H, m, *ArH*), 8.14–8.06 (2H, m, *ArH*), 8.03 (1H, dd, *J* = 7.9, 1.7 Hz, *ArH*), 7.93 (1H, dd, *J* = 8.6, 1.9 Hz, *ArH*), 7.72–7.61 (3H, m, *ArH*), 7.50–7.40 (1H, m, *ArH*), 7.38–7.31 (2H, m, *ArH*), 7.30–7.19 (2H, m, *ArH*), 7.16–7.06 (1H, m, *ArH*), 4.87 (1H, ddd, *J* = 10.3, 7.7, 5.0 Hz, *CH*), 3.69 (3H, s, *OCH_3_*), 3.28–3.09 (2H, m, *CH_2_*). ^13^C NMR (101 MHz, DMSO-*d*_6_) 171.56, 167.79, 164.46, 137.52, 134.69,132.45, 131.88, 129.11, 129.05, 128.63, 128.19, 128.05, 127.81, 127.73, 127.02, 126.52, 123.28, 122.60, 122.53, 121.60, 119.21, 118.99, 115.06, 114.81, 54.31, 51.87, 36.36. HRMS (ESI): calcd for C_28_H_23_FN_2_O_4_+Na: 493.1540 found 493.1543.

Methyl (2-(2-naphthamido)-4-chlorobenzoyl)-L-phenylalaninate **12c**

Compound **11c** (1.0 g, 3 mmol) was treated with naphthoyl chloride (1.5 equivalents), as described in **GP2**, to afford compound **12c** as an off-white fluffy solid (1.3 g, 87% yield).

IR: 3344, 2943, 2111, 1732, 1680, 1589, 1517, 1444, 1396, 1347, 1298, 1219, 11171, 1107, 1171, 1025, 936, 807, 752, 703. ^1^H NMR (300 MHz, DMSO-*d*_6_) 11.97 (1H, s, *NH*), 9.41 (1H, d, *J* = 7.8 Hz, *NH*), 8.63 (1H, d, *J* = 9.0 Hz, *ArH*), 8.45 (1H, d, *J* = 1.8 Hz, *ArH*), 8.16–7.97 (3H, m, *ArH*), 7.92–7.77 (2H, m, *ArH*), 7.73–7.57 (3H, m, *ArH*), 7.35–7.26 (2H, m, *ArH*), 7.22 (2H, t, *J* = 7.6 Hz, *ArH*), 7.11 (1H, d, *J* = 7.3 Hz, *ArH*), 4.81 (1H, ddd, *J* = 10.2, 7.8, 5.2 Hz, *CH*), 3.65 (3H, s, *OCH_3_*), 3.27–2.99 (2H, m, *CH_2_*). ^13^C NMR (101 MHz, DMSO-*d*_6_) 171.56, 167.79, 164.46, 137.52, 134.69,132.45, 131.88, 129.11, 129.05, 128.63, 128.19, 128.05, 127.81, 127.73, 127.02, 126.52, 123.28, 122.60, 122.53, 121.60, 119.21, 118.99, 115.06, 114.81, 54.31, 51.87, 36.36. HRMS (ESI): calcd for C_28_H_23_ClN_2_O_4_+Na: 509.1244 found 5091240.

Methyl (2-(2-naphthamido)-4-bromobenzoyl)-L-phenylalaninate **12d**

Compound **11d** (1 g, 2.7 mmol) was reacted with naphthoyl chloride (1.5 equivalents) according to **GP2** to provide **12d** as a brownish white solid (1.1 g, 80% yield).

IR: 3326, 2943, 1733, 1684, 1587, 1505, 1453, 1392, 1349, 1299, 1220, 1200, 1093, 1025, 913, 827, 751, 702. ^1^H NMR (300 MHz, DMSO-*d*_6_) 11.97 (1H, s, *NH*), 9.42 (1H, d, *J* = 7.8 Hz, *NH*), 8.57 (1H, d, *J* = 8.9 Hz, *ArH*), 8.45 (1H, s, *ArH*), 8.10 (2H, dd, *J* = 9.0, 4.3 Hz, *ArH*), 8.03 (1H, d, *J* = 7.8 Hz, *ArH*), 7.95 (1H, d, *J* = 2.4 Hz, *ArH*), 7.91–7.83 (1H, m, *ArH*), 7.80 (1H, dd, *J* = 8.9, 2.3 Hz, *ArH*), 7.66 (2H, ddd, *J* = 6.7, 4.0, 1.8 Hz, *ArH*), 7.29 (2H, d, *J* = 7.5 Hz, *ArH*), 7.22 (2H, t, *J* = 7.5 Hz, *ArH*), 7.11 (1H, d, *J* = 7.2 Hz, *ArH*), 4.81 (1H, ddd, *J* = 10.1, 7.7, 5.3 Hz, *CH*), 3.65 (3H, s, *OCH_3_*), 3.27 – 3.01 (2H, m, *CH_2_*). ^13^C NMR (101 MHz, DMSO-*d*_6_). 171.56, 167.79, 164.46, 137.52, 134.69,132.45, 131.88, 129.11, 129.05, 128.63, 128.19, 128.05, 127.81, 127.73, 127.02, 126.52, 123.28, 122.60, 122.53, 121.60, 119.21, 118.99, 115.06, 114.81, 54.31, 51.87, 36.36. HRMS (ESI): calcd for C_28_H_23_BrN_2_O_4_+Na: 553.0739 found 553.0732.

(2-(2-Naphthamido)benzoyl)-L-phenylalanine **13a**

As described in **GP3**, compound **12a** (3.5 g, 7.8 mmol) was hydrolysed using LiOH (3.0 equivalents) to give **22a** as a pure white powder in quantitative yield (3.3 g).

IR: 3302, 3025, 1684, 1585, 1506, 1442, 1295, 1219, 912, 859, 808, 752, 687. ^1^H NMR (400 MHz, DMSO-*d*_6_) 12.32 (1H, s, *NH*), 8.98–8.77 (1H, bs, *NH*), 8.64 (1H, dd, *J* = 8.4, 1.2 Hz, *ArH*), 8.47 (1H, d, *J* = 1.8 Hz, *ArH*), 8.10 (2H, dd, *J* = 8.2, 3.7 Hz, *ArH*), 8.02 (1H, dd, *J* = 7.5, 1.8 Hz, *ArH*), 7.90 (1H, dd, *J* = 8.6, 1.9 Hz, *ArH*), 7.74 (1H, dd, *J* = 7.9, 1.5 Hz, *ArH*), 7.70–7.60 (2H, m, *ArH*), 7.57 (1H, ddd, *J* = 8.6, 7.3, 1.5 Hz, *ArH*), 7.33–7.25 (2H, m, *ArH*), 7.25–7.13 (3H, m, *ArH*), 7.09–6.98 (1H, m, *ArH*), 4.60 (1H, t, *J* = 6.7 Hz, *CH*), 3.06 (2H, dd, *J* = 13.7, 9.8 Hz, *CH_2_*). ^13^C NMR (101 MHz, DMSO-*d*_6_) 172.71, 168.79, 164.47, 139.16, 137.97, 134.45, 132.46, 132.23, 131.83, 129.14, 129.07, 128.71, 128.37, 128.16, 127.79, 127.72, 127.10, 126.40, 123.23, 122.90, 120.36, 120.24, 54.14, 36.19. HRMS (ESI): calcd for C_27_H_22_N_2_O_4_+Na: 461.1477 found 461.1471.

(2-(2-Naphthamido)-4-fluorobenzoyl)-L-phenylalanine **13b**

Compound **12b** (1.0 g, 2.1 mmol) was treated with LiOH (3.0 equivalents), according to **GP3**, to afford **13b** as a white powder in quantitative yield (0.97 g).

IR: 3061, 2601, 2341, 1730, 1649, 1605, 1519, 1413, 1309, 1206, 949, 823, 756, 699. ^1^H NMR (400 MHz, DMSO-*d*_6_) 11.99 (1H, s, NH), 9.19 (1H, d, *J* = 8.1 Hz, NH), 8.62 (1H, dd, *J* = 9.2, 5.3 Hz, ArH), 8.45 (1H, d, *J* = 1.8 Hz, ArH), 8.10 (2H, d, *J* = 8.5 Hz, ArH), 8.03 (1H, dd, *J* = 7.7, 1.8 Hz, ArH), 7.87 (1H, dd, *J* = 8.6, 1.8 Hz, ArH), 7.72–7.56 (3H, m, ArH), 7.49 (1H, ddd, *J* = 9.2, 8.0, 3.0 Hz, ArH), 7.36–7.27 (2H, m, ArH), 7.21 (2H, t, *J* = 7.6 Hz, ArH), 7.14–6.98 (1H, m, ArH), 4.79–4.64 (1H, m, CH), 3.05 (2H, m, CH_2_). ^13^C NMR (101 MHz, DMSO-*d*_6_) 172.48, 167.37, 164.45, 137.91, 135.60, 134.45, 132.20, 131.62, 129.14, 129.05, 128.70, 128.18, 128.14, 127.82, 127.72, 127.11, 126.39, 123.21, 119.03, 114.96, 54.29, 36.28. HRMS (ESI): calcd for C_27_H_21_FN_2_O_4_+Na: 479.1383 found 479.1378.

(2-(2-Naphthamido)-4-chlorobenzoyl)-L-phenylalanine **13c**

According to **GP3**, **12c** (1.1 g, 2.3 mmol) was hydrolysed using LiOH (3.0 equivalents) to give **13c** in quantitative yield (1.0 g).

IR: 3313, 2935, 2596, 1726, 1665, 1588, 1521, 1454, 1393, 1308, 1225, 1094, 1014, 973, 913, 812, 759, 703. ^1^H NMR (300 MHz, DMSO-*d*_6_) 12.20 (1H, s, *NH*), 9.04 (1H, s, *NH*), 8.64 (1H, d, *J* = 9.0 Hz, *ArH*), 8.45 (1H, d, *J* = 1.8 Hz, *ArH*), 8.14 – 8.05 (2H, m, *ArH*), 8.04–7.97 (1H, m, *ArH*), 7.87 (1H, dd, *J* = 8.6, 1.8 Hz, *ArH*), 7.80 (1H, d, *J* = 2.5 Hz, *ArH*), 7.71–7.57 (3H, m, *ArH*), 7.34–7.24 (2H, m, *ArH*), 7.16 (2H, t, *J* = 7.6 Hz, *ArH*), 7.06–6.94 (1H, m, *ArH*), 4.60 (1H, s, *CH*), 3.31–2.93 (4H, m, *CH_2_*). ^13^C NMR (101 MHz, DMSO-*d*_6_) 172.46, 167.35, 164.43, 137.89, 135.58, 134.43, 132.18, 131.60, 129.12, 129.03, 128.68, 128.16, 128.12, 127.80, 127.70, 127.09, 126.37, 123.19, 119.01, 114.94, 54.27, 36.26. HRMS (ESI): calcd for C_27_H_21_ClN_2_O_4_+Na: 495.1088 found 495.1091

(2-(2-Naphthamido)-4-bromobenzoyl)-L-phenylalanine **13d**

Compound **12d** (1.0 g, 1.9 mmol) was reacted with LiOH (3.0 equivalents) to give **13d** as a pure brownish solid in quantitative yield (0.97 g).

IR: 3314, 2935, 2602, 2342, 1728, 1665, 1589, 1521, 1454, 1393, 1307, 1224, 1093, 1014, 973, 913, 813, 758, 703. ^1^H NMR (300 MHz, DMSO-*d*_6_) 12.06 (1H, s, *NH*), 9.31 (1H, d, *J* = 8.2 Hz, *NH*), 8.58 (1H, d, *J* = 8.9 Hz, *ArH*), 8.44 (1H, d, *J* = 1.8 Hz, *ArH*), 8.10 (2H, d, *J* = 8.4 Hz, *ArH*), 8.06–8.00 (1H, m, *ArH*), 7.96 (1H, d, *J* = 2.3 Hz, *ArH*), 7.86 (1H, dd, *J* = 8.6, 1.8 Hz, *ArH*), 7.79 (1H, dd, *J* = 9.0, 2.3 Hz, *ArH*), 7.66 (2H, ddd, *J* = 6.9, 4.5, 1.8 Hz, *ArH*), 7.34–7.26 (2H, m, *ArH*), 7.20 (2H, t, *J* = 7.6 Hz, *ArH*), 7.11–7.03 (1H, m, *ArH*), 4.75 (1H, ddd, *J* = 10.6, 7.9, 4.5 Hz, *CH*), 3.18–2.95 (2H, m, *CH_2_*). ^13^C NMR (101 MHz, DMSO-*d*_6_) 172.43, 167.32, 164.40, 137.86, 135.55, 134.40, 132.15, 131.57, 129.09, 129.00, 128.65, 128.13, 128.09, 127.77, 127.67, 127.06, 126.34, 123.16, 118.98, 114.91, 54.24, 36.23. HRMS (ESI): calcd for C_27_H_21_BrN_2_O_4_+Na: 539.0582 found 539.0586

(*S*)-*N*-(2-((1-((2-aminoethyl)amino)-1-oxo-3-phenylpropan-2-yl)carbamoyl)phenyl)-2-naphthamide **1a**

The amide coupling reaction between **13a** (0.7 g,1.6 mmol) and *tert*-butyl (2-aminoethyl)carbamate (1.1 equivalents), according to **GP3**, gave off-white solid (0.5 g, 54% yield). Subsequently, the product (0.4 g, 6.9 mmol) was then treated with TFA (2 equivalents) at 0 °C for 1 hour, according to **GP5**. The resulting white solid underwent a NaHCO_3_ wash to afford **1a** in quantitative yield (0.33 g).

IR: 3295, 2928, 1657, 1587, 1516, 1444, 1301, 1177, 1128, 955, 911, 835, 754, 698. ^1^H NMR (400 MHz, DMSO-*d*_6_) 12.05 (1H, s, *NH*), 9.02 (1H, d, *J* = 8.4 Hz, *NH*), 8.54 (1H, dd, *J* = 8.4, 1.2 Hz, *ArH*), 8.45 (1H, m, *NH*), 8.36 (1H, t, *J* = 5.7 Hz, *ArH*), 8.13–7.97 (3H, m, *ArH*), 7.88–7.83 (1H, m, *ArH*), 7.81–7.77 (1H, m, *ArH*), 7.70–7.64 (3H, m, *ArH*), 7.64–7.55 (1H, m, *ArH*), 7.36–7.26 (2H, m, *ArH*), 7.27–7.14 (3H, m, *ArH*), 7.10–6.98 (1H, m, *ArH*), 4.79 (ddd, *J* = 10.7, 8.4, 4.3 Hz, *CH*), 3.26–2.92 (4H, m, *CH_2_*), 2.84 (2H, t, *J* = 6.7 Hz, *CH_2_*), 1.52 (2H, bs, *NH_2_*). ^13^C NMR (101 MHz, DMSO) 171.48, 168.61, 164.54, 138.87, 138.15, 134.43, 132.28, 132.21, 131.83, 129.10, 128.61, 128.53, 128.18, 128.06, 127.88, 127.75, 127.14, 126.30, 123.27, 122.97, 121.09, 120.60, 54.68, 38.45, 37.04, 36.59. HRMS (ESI): calcd for C_29_H_28_N_4_O_3_+Na: 503.2059 found 503.2054.

(*S*)-*N*-(2-((1-((3-aminopropyl)amino)-1-oxo-3-phenylpropan-2-yl)carbamoyl)phenyl)-2-naphthamide **2a**

According to **GP4**, compound **13a** (1.0 g, 2.3 mmol) was reacted with *tert*-butyl (2-aminoethyl)carbamate (1.1 equivalents) followed by TFA deprotection as described in GP5 and NaHCO_3_ wash to afford **2a** as a white solid (0.54 g, 50% yield).

IR: 3285, 3050, 2944, 1652, 1590, 1506, 1441, 1389, 1291, 1181, 1129, 1046, 1033, 913, 836. ^1^H NMR (400 MHz, DMSO-*d*_6_) 12.11 (1H, s, *NH*), 9.06 (1H, d, *J* = 8.3 Hz, *NH*), 8.55 (1H, d, *J* = 8.4 Hz, *ArH*), 8.46 (1H, d, *J* = 1.8 Hz, *NH*), 8.34 (1H, t, *J* = 5.8 Hz, *ArH*), 8.09 (2H, d, *J* = 8.3 Hz, *ArH*), 8.04 (1H, dd, *J* = 7.4, 1.9 Hz, *ArH*), 7.88 (1H, dd, *J* = 8.6, 1.8 Hz, *ArH*), 7.79 (1H, dd, *J* = 8.0, 1.5 Hz, *ArH*), 7.67 (1H, m, *ArH*), 7.58 (1H, td, *J* = 8.2, 7.8, 1.5 Hz, *ArH*), 7.38–7.30 (2H, m, *ArH*), 7.26–7.15 (3H, m, *ArH*), 7.11–7.02 (1H, m, *ArH*), 4.75 (1H, ddd, *J* = 10.7, 8.3, 4.4 Hz, *CH*), 3.22–2.92 (4H, m, *CH_2_*), 2.85–2.61 (2H, m, *CH_2_*), 1.66 (2H, m, *CH_2_*), 1.52 (2H, bs, *NH_2_*). ^13^C NMR (101 MHz, DMSO) 171.21, 168.65, 164.54, 138.92, 138.13, 134.42, 132.28, 132.20, 131.84, 129.09, 129.07, 128.60, 128.16, 128.07, 127.87, 127.74, 127.12, 126.32, 123.25, 122.95, 121.02, 120.58, 54.91, 37.17, 36.71, 35.74, 27.35. HRMS (ESI): calcd for C_30_H_30_N_4_O_3_+Na: 517.2216 found 517.2214.

(S)-N-(2-((1-((3-guanidinopropyl)amino)-1-oxo-3-phenylpropan-2-yl)carbamoyl)-phenyl)-2-naphthamide **5a**

The primary amine **2a** (0.28 g, 0.57 mmol) was dissolved in anhydrous DCM under nitrogen atmosphere. *N*, *N'*-Di-Boc-1H-pyrazole-1-carboxamidine (1.1. equivalent) and trimethylamine (2.0 equivalent) were added to the reaction mixture. After completion of the reaction (18 h), the reaction mixture was removed under reduced pressure and the resulting crude material was subjected to column chromatography with DCM: MeOH (5%) as the mobile phase. Subsequently, the pure white solid was treated with TFA according to **GP5**. The resulting solid was dissolved in saturated NaHCO_3_ (20 mL) and stirred for 30 minutes. Milli-Q water (30 mL) was added and the reaction mixture was extracted with ethyl acetate (3 x 15 mL). The resulting organic phase was dried over Na_2_SO_4_, filtered, and concentrated under reduce pressure to afford **5a** as an off-white solid in quantitative yield (0.21 g).

IR: 3270, 3035, 2937, 2343, 2113, 1735, 1687, 1576, 1512, 1432, 1273, 1204, 1162, 1062, 917, 840, 753. ^1^H NMR (400 MHz, DMSO-*d*_6_) 11.25 (1H, s, *NH*), 8.84 (1H, d, *J* = 8.2 Hz, *NH*), 8.37 (1H, dd, *J* = 8.4, 1.2 Hz, *ArH*), 8.28–8.21 (1H, m, *ArH*), 8.11 (1H, t, *J* = 5.6 Hz, *NH*), 8.05 (1H, t, *J* = 5.9 Hz, *ArH*), 7.86 (2H, dd, *J* = 9.1, 2.2 Hz, *ArH*), 7.84 (1H, bs, *NH*), 7.81 (1H, dd, *J* = 7.6, 1.8 Hz, *ArH*), 7.67 (1H, dd, *J* = 8.6, 1.8 Hz, *ArH*), 7.58 (1H, dd, *J* = 8.0, 1.5 Hz, *ArH*), 7.50–7.42 (2H, m, *ArH*), 7.42–7.32 (1H, m, *ArH*), 7.19–7.11 (m, 2H), 7.01 (t, *J* = 7.5 Hz, 2H), 6.99 (2H, m, *NH*), 6.91–6.83 (1H, m, *ArH*), 4.52 (1H, ddd, *J* = 10.4, 8.1, 4.6 Hz, *CH*), 3.07–2.74 (6H, m, *CH_2_*), 2.64 (1H, bs, *NH*), 1.47–1.30 (2H, m, *CH_2_*). ^13^C NMR (101 MHz, DMSO-*d*_6_) 170.81, 170.73, 168.74, 168.67, 155.32, 151.95, 139.01, 138.95, 138.27, 134.46, 132.23, 131.87, 129.18, 129.08, 128.64, 128.58, 128.09, 128.07, 127.84, 127.78, 127.74, 127.13, 127.07, 126.32, 123.28, 122.94, 121.12, 121.03, 120.53, 82.83, 78.16, 67.00, 55.18, 55.03, 54.93, 31.33, 28.00, 27.63. HRMS (ESI): calcd for C_31_H_32_N_6_O_3_+Na: 559.2434 found 559.2330.

(*S*)-*N*-(2-((1-((3-(dimethylamino)propyl)amino)-1-oxo-3-phenylpropan-2-yl)carbamoyl)-phenyl)-2-naphthamide **3a**

Compound **13a** (1.5 g, 3.4 mmol) underwent amide coupling reaction with *N*^1^,*N*^1^-dimethylpropane-1,3-diamine (1.1 equivalents) according to **GP4** to afford **3a** as a pure white solid (1.3 g, 78% yield).

IR: 3289, 3234, 3056, 2935, 2622, 2485, 2343, 2114, 1935, 1656, 1587, 1503, 1432, 1287, 1234, 1028, 910, 859, 751. ^1^H NMR (400 MHz, DMSO-*d*_6_) 12.13 (1H, s, *NH*), 9.05 (1H, d, *J* = 8.4 Hz, *NH*), 8.54 (1H, dd, *J* = 8.4, 1.2 Hz, *ArH*), 8.46 (1H, d, *J* = 1.8 Hz, *ArH*), 8.26 (1H, t, *J* = 5.7 Hz, *NH*), 8.09 (2H, d, *J* = 8.2 Hz, *ArH*), 8.03 (1H, dd, *J* = 7.5, 1.9 Hz, *ArH*), 7.88 (1H, dd, *J* = 8.6, 1.8 Hz, *ArH*), 7.79 (1H, dd, *J* = 7.9, 1.5 Hz, *ArH*), 7.66 (2H, tt, *J* = 7.0, 5.3 Hz, *ArH*), 7.58 (1H, td, *J* = 8.4, 7.9, 1.5 Hz, *ArH*), 7.42–7.29 (2H, m, *ArH*), 7.28–7.15 (3H, m, *ArH*), 7.13–6.97 (1H, m, *ArH*), 4.73 (1H, ddd, *J* = 10.7, 8.3, 4.4 Hz, *CH*), 3.18–2.96 (4H, m, *CH_2_*), 2.43 (2H, t, *J* = 7.5 Hz, *CH_2_*), 2.23 (6H, s, *CH_3_*), 1.59 (2H, td, *J* = 7.0, 3.0 Hz, *CH_2_*). ^13^C NMR (101 MHz, DMSO) 170.59, 168.62, 164.55, 138.92, 138.32, 134.43, 132.21, 131.84, 129.13, 129.06, 128.60, 128.53, 128.13, 128.04, 127.82, 127.73, 127.08, 126.26, 123.29, 122.93, 121.11, 120.56, 56.22, 55.02, 37.12, 36.89, 26.36. HRMS (ESI): calcd for C_32_H_34_N_4_O_3_+Na: 545.2529 found 545.2523.

(*S*)-*N*-(2-((1-((3-(dimethylamino)propyl)amino)-1-oxo-3-phenylpropan-2-yl)carbamoyl)-5-fluorophenyl)-2-naphthamide **6a**

According to **GP4**, compound **13b** (0.9 g, 2.0 mmol) was reacted with *N*^1^, *N*^1^-dimethylpropane-1,3-diamine (1.1 equivalents) to give **6a** as a pure white solid (0.78 g, 72% yield).

IR: 3292, 3230, 3055, 2950, 2609, 2478, 2343, 2114, 1657, 1601, 1521, 1411, 1300, 1241, 1203, 1099, 945, 871, 824, 754, 677. ^1^H NMR (400 MHz, DMSO-*d*_6_) 11.89 (1H, s, *NH*), 9.16 (1H, d, *J* = 8.4 Hz, *NH*), 8.50 (1H, dd, *J* = 9.2, 5.3 Hz, *ArH*) 8.45 (1H, d, *J* = 1.8 Hz, *ArH*), 8.31 (1H, t, *J* = 5.8 Hz, *NH*), 8.16–8.05 (2H, m, *ArH*), 8.03 (1H, dd, *J* = 7.7, 1.9 Hz, *ArH*), 7.87 (1H, dd, *J* = 8.6, 1.8 Hz, *ArH*), 7.76–7.56 (3H, m, *ArH*), 7.47 (1H, ddd, *J* = 9.2, 8.0, 3.0 Hz, *ArH*), 7.38–7.29 (2H, m, *ArH*), 7.20 (2H, t, *J* = 7.6 Hz, *ArH*), 7.13–6.99 (1H, m, *ArH*), 4.73 (1H, ddd, *J* = 10.8, 8.3, 4.5 Hz, *CH*), 3.24–3.15 (2H, m, *CH_2_*), 3.14–2.91 (2H, m, *CH_2_*), 2.61 (2H, d, *J* = 13.7, 10.7 Hz, *CH_2_*), 2.39 (6H, s, *CH_3_*), 1.65 (2H, td, *J* = 7.1, 2.7 Hz, CH_2_). ^13^C NMR (101 MHz, DMSO-*d*_6_) 170.60, 167.36, 167.34, 164.57, 158.32, 155.93, 138.16, 135.25, 135.23, 134.44, 132.18, 131.64, 129.10, 129.06, 128.60, 128.16, 128.05, 127.87, 127.73, 127.11, 126.31, 123.28, 123.15, 123.08, 122.93, 118.95, 118.74, 115.30, 115.06, 55.23, 55.01, 48.59, 43.17, 37.11, 36.33, 25.16. HRMS (ESI): calcd for C_32_H_33_FN_4_O_3_+Na: 563.2434 found 563.2431.

(*S*)-*N*-(5-chloro-2-((1-((3-(dimethylamino)propyl)amino)-1-oxo-3-phenylpropan-2-yl)carbamoyl)phenyl)-2-naphthamide **7a**

Compound **13c** (0.90 g, 1.9 mmol) was subjected to amide coupling reaction with *N*^1^,*N*^1^-dimethylpropane-1,3-diamine (1.1 equivalents), according to **GP4**, to give **7a** as a pure off-white solid (0.78 g, 74% yield).

IR: 3290, 3053, 2928, 2170, 1654, 1583, 1505, 1442, 1399, 1301, 1089, 913, 811, 755, 702. ^1^H NMR (300 MHz, DMSO-*d*_6_) 11.97 (1H, s, *NH*), 9.22 (1H, d, *J* = 8.3 Hz, *NH*), 8.53 (1H, d, *J* = 8.9 Hz, *ArH*), 8.44 (1H, d, *J* = 1.8 Hz, *ArH*), 8.31 (1H, t, *J* = 5.8 Hz, *NH*), 8.09 (2H, d, *J* = 8.5 Hz, *ArH*), 8.06–7.97 (1H, m, *ArH*), 7.91–7.77 (2H, m, *ArH*), 7.74–7.57 (3H, m, *ArH*), 7.32 (2H, d, *J* = 7.2 Hz, *ArH*), 7.19 (2H, t, *J* = 7.5 Hz, *ArH*), 7.05 (1H, t, *J* = 7.3 Hz, *ArH*), 4.73 (1H, ddd, *J* = 10.7, 8.2, 4.5 Hz, *CH*), 3.22–2.89 (4H, m, *CH_2_*), 2.63 (2H, t, *J* = 7.5 Hz, *CH_2_*), 2.40 (6H, s, *CH_3_*), 1.75–1.56 (2H, m, *CH_2_*). ^13^C NMR (76 MHz, DMSO-*d*_6_) 170.65, 167.37, 164.66, 138.15, 137.70, 134.51, 132.19, 131.90, 131.54, 129.12, 128.68, 128.29, 128.07, 127.96, 127.77, 127.19, 126.80, 126.35, 123.26, 122.82, 122.40, 55.28, 55.00, 43.22, 37.12, 36.35.HRMS (ESI): calcd for C_32_H_33_ClN_4_O_3_+Na: 579.2139 found 579.2132.

(*S*)-*N*-(5-bromo-2-((1-((3-(dimethylamino)propyl)amino)-1-oxo-3-phenylpropan-2-yl)carbamoyl)phenyl)-2-naphthamide **8a**

According to **GP4**, compound **13d** (0.9 g, 1.7 mmol) was reacted with *N*^1^,*N*^1^-dimethylpropane-1,3-diamine (1.1 equivalents) to give **8a** as a brownish white solid (0.73 g, 70% yield).

IR: 3296, 3055, 2927, 1654, 1583, 1505, 1441, 1395, 1303, 1090, 912, 812, 755, 704. ^1^H NMR (300 MHz, DMSO-*d*_6_) 11.97 (1H, s, *NH*), 9.22 (1H, d, *J* = 8.3 Hz, *NH*), 8.50–8.41 (2H, m, *ArH*), 8.31 (1H, t, *J* = 5.8 Hz, *NH*), 8.12–8.06 (2H, m, *ArH*), 8.06–8.01 (1H, m, *ArH*), 7.96 (1H, d, *J* = 2.3 Hz, *ArH*), 7.85 (1H, dd, *J* = 8.6, 1.8 Hz, *ArH*), 7.77 (1H, dd, *J* = 8.9, 2.3 Hz, *ArH*), 7.73–7.57 (2H, m, *ArH*), 7.38–7.26 (2H, m, *ArH*), 7.19 (2H, dd, *J* = 8.3, 6.9 Hz, *ArH*), 7.11–6.98 (1H, m, *ArH*), 4.73 (1H, td, *J* = 9.4, 8.3, 4.5 Hz, *CH*), 3.24–2.88 (4H, m, *CH_2_*), 2.63–2.69 (2H, m, *CH_2_*), 2.44 (6H, s, *CH_3_*), 1.74–1.53 (2H, m, *CH_2_*). ^13^C NMR (76 MHz, DMSO-*d*_6_) 170.70, 167.29, 164.66, 138.19, 138.11, 134.78, 134.52, 132.19, 131.55, 131.16, 129.17, 129.13, 128.70, 128.28, 128.06, 127.98, 127.79, 127.20, 126.34, 123.27, 123.10, 122.64, 114.78, 55.02, 42.86, 37.16, 36.28, 24.87. HRMS (ESI): calcd for C_32_H_33_BrN_4_O_3_+Na: 623.1634 found 623.1629.

(*S*)-3-(2-(2-(2-naphthamido)benzamido)-3-phenylpropanamido)-*N*,*N*,*N*-trimethylpropan-1-aminium iodide **4a**

Compound **3a** (0.3 g, 0.6 mmol) was suspended in anhydrous THF under argon atmosphere. After cooling to 0 °C, methyl iodide (2.0 equivalents) was added dropwise and the reaction mixture was stirred at room temperature for 16 h. After completion, the reaction mixture was concentrated under reduced pressure to remove solvents. The resulting crude material was washed with cold DCM to afford hydrogelator **4a** as a pure yellowish white solid (0.29 g, 95% yield).

IR: 3251, 3033, 2940, 2620, 1657,1597, 1510, 1442, 1304, 1119, 960, 911, 824, 751.  ^1^H NMR (300 MHz, DMSO-*d*_6_) 12.10 (1H, s, *NH*), 9.62 (1H, bs, *NH*), 9.06 (1H, d, *J* = 8.3 Hz, *NH*), 8.54 (1H, d, *J* = 8.3 Hz, *ArH*), 8.46 (1H, d, *J* = 1.8 Hz, *ArH*), 8.09 (2H, d, *J* = 8.4 Hz, *ArH*, 8.06–7.99 (1H, m, *ArH)*, 7.88 (1H, dd, *J* = 8.6, 1.8 Hz, *ArH*), 7.80 (1H, dd, *J* = 8.0, 1.5 Hz, *ArH*), 7.72–7.63 (2H, m, *ArH*), 7.63–7.51 (1H, m, *ArH*), 7.34 (2H, d, *J* = 7.2 Hz, *ArH*), 7.22 (3H, td, *J* = 7.6, 3.0 Hz, *ArH*), 7.07 (1H, t, *J* = 7.3 Hz, *ArH)*, 4.84–4.66 (1H, m, *CH*), 3.26–2.85 (6H, m, *CH_2_*), 2.67 (9H, s, *CH_3_*), 1.73 (2H, m, *CH_2_*). ^13^C NMR (76 MHz, DMSO-*d*_6_) 170.59, 168.62, 164.55, 138.92, 138.32, 134.43, 132.21, 131.84, 129.13, 129.06, 128.60, 128.53, 128.13, 128.04, 127.82, 127.73, 127.08, 126.26, 123.29, 122.93, 121.11, 120.56, 56.22, 55.02, 37.12, 36.89, 26.36. HRMS (ESI): calcd for C_33_H_37_N_4_O_3_: 537.2860 found 537.2858.

(*S*)-3-(2-(2-(2-naphthamido)benzamido)-3-phenylpropanamido)-*N*,*N*-dimethylpropan-1-aminium trifluoroacetate **9a**

Compound **3a** (0.25 g, 0.48 mmol) was dissolved in DCM and cooled to 0 °C. TFA (1.5 equivalent) was added and the reaction mixture was stirred for 30 minutes. Subsequently this mixture was concentrated under reduced pressure. The resulting pale brown crude material was washed with diethyl ether (2 x 15 mL), filtered, and dried to afford **9a** as an off-white solid in quantitative yield (0.35 g).

IR: 3287, 3056, 2955, 2721, 2649, 2343, 2121, 1670, 1587, 1507, 1442, 1300, 1173, 1123, 980, 912, 798, 750, 698.  ^1^H NMR (300 MHz, DMSO-*d*_6_) 12.10 (1H, s, *NH*), 9.62 (1H, bs, *NH*), 9.06 (1H, d, *J* = 8.3 Hz, *NH*), 8.54 (1H, d, *J* = 8.3 Hz, *ArH*), 8.46 (1H, d, *J* = 1.8 Hz, *ArH*), 8.36 (1H, t, *J* = 5.8 Hz, *NH*), 8.09 (2H, d, *J* = 8.4 Hz, *ArH*, 8.06 – 7.99 (1H, m, *ArH)*, 7.88 (1H, dd, *J* = 8.6, 1.8 Hz, *ArH*), 7.80 (1H, dd, *J* = 8.0, 1.5 Hz, *ArH*), 7.72–7.63 (2H, m, *ArH*), 7.63–7.51 (1H, m, *ArH*), 7.34 (2H, d, *J* = 7.2 Hz, *ArH*), 7.22 (3H, td, *J* = 7.6, 3.0 Hz, *ArH*), 7.07 (1H, t, *J* = 7.3 Hz, *ArH)*, 4.84–4.66 (1H, m, *CH*), 3.26–2.85 (6H, m, *CH_2_*), 2.66 (6H, s, *CH_3_*), 1.73 (2H, m, *CH_2_*). ^13^C NMR (101 MHz, DMSO-*d*_6_) 170.57, 168.60, 164.53, 138.90, 138.30, 134.41, 132.19, 131.82, 129.11, 129.04, 128.58, 128.51, 128.11, 128.02, 127.80, 127.71, 127.06, 126.24, 123.27, 122.91, 121.09, 120.54, 56.20, 55.00, 37.10, 36.87, 26.34. HRMS (ESI): calcd for C_32_H_34_N_4_O_3_+H 523.2704 found 523.2711.

(*S*)-3-(2-(2-(2-naphthamido)benzamido)-3-phenylpropanamido)-*N*,*N*-dimethylpropan-1-aminium chloride **10a**

 Compound **3a** (0.3 g, 0.57 mmol) was dissolved in DCM and the solution cooled down to 0 °C and 4M HCl in dioxane (1.5 equivalent) was added dropwise. The reaction mixture was stirred at room temperature for 1 hour. The resulting precipitate was filtered, dried, and washed with diethyl ether to provided **10a** as a white solid in quantitative yield (0.31 g).

IR: 3284, 3232, 3053, 2933, 2621, 2485, 2343, 2114, 1935, 1656, 1587, 1503, 1432, 1287, 1234, 1028, 910, 859,670. ^1^H NMR (400 MHz, DMSO-*d*_6_) 12.00 (1H s, *NH*), 9.52 (1H, s, *NH*), 8.96 (1H, d, *J* = 8.3 Hz, *NH*), 8.44 (1H, d, *J* = 8.3 Hz, *ArH*), 8.36 (1H, d, *J* = 1.8 Hz, *ArH*), 8.26 (1H, t, *J* = 5.8 Hz, *NH*), 7.99 (2H, d, *J* = 8.4 Hz, *ArH*), 7.96–7.89 (1H, m, *ArH*), 7.78 (1H, dd, *J* = 8.6, 1.8 Hz, *ArH*), 7.70 (1H, dd, *J* = 8.0, 1.5 Hz, *ArH*), 7.62–7.51 (2H, m, *ArH*), 7.52–7.42 (1H, m, *ArH*), 7.24 (2H, d, *J* = 7.2 Hz, *ArH*), 7.12 (3H, td, *J* = 7.5, 3.0 Hz, *ArH*), 6.97 (1H, t, *J* = 7.3 Hz, *ArH*), 4.65 (1H, ddd, *J* = 10.5, 8.1, 4.5 Hz, CH), 3.18–2.71 (6H, m, *CH_2_*), 2.56 (6H, s, *CH_3_*), 1.70–1.49 (2H, m, *CH_2_*). ^13^C NMR (101 MHz, DMSO-*d*_6_) 170.55, 168.58, 164.51, 138.88, 138.28, 134.39, 132.17, 131.80, 129.09, 129.02, 128.56, 128.49, 128.09, 128.00, 127.78, 127.69, 127.04, 126.22, 123.25, 122.89, 121.07, 120.52, 56.18, 54.98, 37.08, 36.85, 26.32. HRMS (ESI): calcd for C_32_H_34_N_4_O_3_+H 523.2704 found 523.2711.

1. Aldilla, V. R.; Chen, R.; Martin, A. D.; Marjo, C. E.; Rich, A. M.; Black, D. S.; Thordarson, P.; Kumar, N., Anthranilamide-based Short Peptides Self-Assembled Hydrogels as Antibacterial Agents. *Sci. Rep.* **2020,** *10* (1), 1-12.
